# Galectin-9 in cancer therapy: from immune checkpoint ligand to promising therapeutic target

**DOI:** 10.3389/fcell.2023.1332205

**Published:** 2024-01-09

**Authors:** Minpu Zhang, Cun Liu, Ye Li, Huayao Li, Wenfeng Zhang, Jingyang Liu, Liquan Wang, Changgang Sun

**Affiliations:** ^1^ College of First Clinical Medicine, Shandong University of Traditional Chinese Medicine, Jinan, China; ^2^ College of Traditional Chinese Medicine, Weifang Medical University, Weifang, China; ^3^ Faculty of Chinese Medicine and State Key Laboratory of Quality Research in Chinese Medicines, Macau University of Science and Technology, Macau, China; ^4^ Department of Thyroid and Breast Surgery, Weifang People’s Hospital, Weifang, China; ^5^ Department of Oncology, Weifang Traditional Chinese Hospital, Weifang, China

**Keywords:** galectin-9, pan-cancer, biological functions, therapeutic target, immunotharapy

## Abstract

Galectin-9 (Gal-9) is a vital member of the galectin family, functioning as a multi-subtype galactose lectin with diverse biological roles. Recent research has revealed that Gal-9’s interaction with tumors is an independent factor that influences tumor progression. Furthermore, Gal-9 in the immune microenvironment cross-talks with tumor-associated immune cells, informing the clarification of Gal-9’s identity as an immune checkpoint. A thorough investigation into Gal-9’s role in various cancer types and its interaction with the immune microenvironment could yield novel strategies for subsequent targeted immunotherapy. This review focuses on the latest advances in understanding the direct and indirect cross-talk between Gal-9 and hematologic malignancies, in addition to solid tumors. In addition, we discuss the prospects of Gal-9 in tumor immunotherapy, including its cross-talk with the ligand TIM-3 and its potential in immune-combination therapy.

## 1 Introduction

**FIGURE 1:**
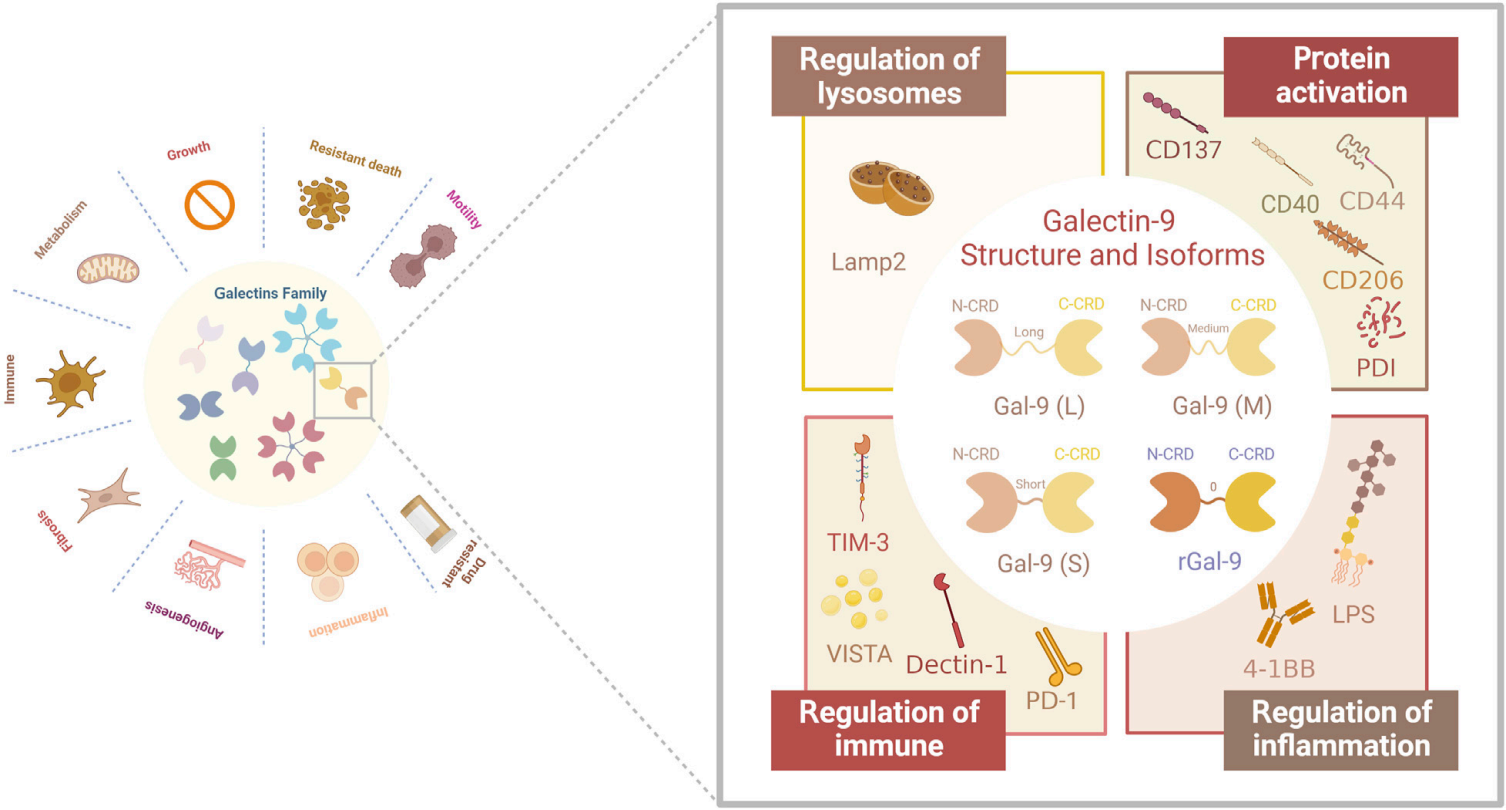
Structure and structural domains of Gal-9 and their biological functions associated with different receptors. Gal-9 has been found in three natural Gal-9 isoforms with different interdomain linker lengths (long, medium and short). In addition, a recombinant form of Gal-9 with a linker length of two amino acids has been produced. In addition, the receptors for Gal-9 are complex and diverse: lysosomal-associated membrane protein 2 (LAMP2), TNF receptor superfamily member CD137, CD44 adhesion molecule, CD40, macrophage marker CD206, protein disulfide bond isomerase (PDI) involved in T cell migration, immune receptors PD-1, TIM-3 and VISTA, Dectin-1, which are clinically relevant, in addition to bacterial lipopolysaccharide (LPS), 4-1BB, which is associated with inflammatory responses.

Galectins (Gals) are a family of lectins defined by a shared amino acid sequence. Their structure features a carbohydrate recognition domain (CRD) that specifically binds to polysaccharides containing *ß*-galactoside. This structural feature serves as a foundation for understanding the function of such sugar chain-binding proteins (Nagae et al., 2008). Apart from recognizing the typical glycan structure, each member of the Gals family exhibits preferential binding to specific glycosylated proteins and/or lipids found on the cell surface or in the extracellular matrix (Barondes et al., 1994a; Barondes et al., 1994b). This specific binding influences the interaction between Gals and human cells. There are 16 identified Gals that exert both intracellular and extracellular effects. They are involved in various biological functions, including the regulation of cell growth, apoptosis, pre-mRNA splicing, cell-cell and cell-matrix adhesion, cell polarity, and innate/acquired immunity. These functions may be linked to the relationship between Gals and glycated proteins and/or lipids (The figure is depicted in Figure 1) (Chou et al., 2018). The widespread distribution of Gals in most organs of the body likely contributes to their broad range of biological functions and their ability to interact with various target cells.

Recent articles have shed light on the involvement of Gals in tumors, where alterations in their expression levels are closely linked to tumor biology. Aberrant expression of Gals has been implicated in tumorigenesis, progression, and metastasis, making several members of the family potential prognostic markers for various types of cancer (Kapetanakis and Busson, 2023). Galectin-9 (Gal-9), as a member of the Gals family, is a soluble molecule with broad availability and noteworthy significance due to its diverse biological functions and potent immunomodulatory effects. Gal-9 was first discovered as an effective eosinophil chemoattractant and is widely distributed in liver, small intestine, thymus, lung, spleen, kidney and other organs (Wada and Kanwar, 1997). In addition, research by Hirashima M et al. found that Gal-9 cannot be detected in endothelial cells, fibroblasts and astrocytes under physiological conditions, but some cytokines (such as IFN-γ or IL-1β) can be upregulated (Hirashima et al., 2002). The expression of Gal-9 indicates that the general biological function and regulatory mechanism of Gal-9 are different from those under pathological conditions. The characteristic regulation of alternative splicing in Gal-9 gives rise to a wide range of biological functions in different cell types, playing a pivotal role in shaping cellular phenotypes and physiological processes (Marasco and Kornblihtt, 2023). For example, in endothelial cell biology, isoforms of Gal-9 can influence the activation of resting endothelial cells and the migration of activated endothelial cells. While distinct effects can be observed *in vitro*, this study shows that all isoforms of Gal-9 can hinder angiogenesis *in vivo* (Aanhane et al., 2018). Since alternative splicing occurs, Gal-9 manifests in three isoforms with varying lengths of linking peptides, and different cell types can express one, two, or all three isoforms (Yasinska et al., 2019). Notably, Gal-9 exhibits unique activity profiles, acting as a “double-edged sword” depending on the cellular localization of its binding partners. Intracellular Gal-9, for example, stimulates the expression of pro-inflammatory cytokines in monocytes by binding to the transcription factor NF-IL6, while extracellular Gal-9 triggers monocyte death.

The diverse range of biological functions exhibited by Gal-9 has resulted in various impacts on tumors (Chou et al., 2018). In certain types of cancers, such as breast cancer (Yasinska et al., 2019), nasopharyngeal carcinoma (Chen et al., 2017), and melanoma (Kageshita et al., 2002), high expression of Gal-9 has been associated with cancer cell aggregation, while low expression is linked to cell invasion. In cases of carcinoma *in situ*, Gal-9’s high expression facilitates communication between cells and the extracellular matrix, whereas low expression in metastatic tumors suggests a potential inhibitory role in tumor metastasis. For example, in breast cancer, previous reports have suggested that Gal-9 exhibits anti-metastatic properties (Irie et al., 2005; Yamauchi et al., 2006). Furthermore, Gal-9 demonstrates anti-proliferative abilities in malignant tumors, particularly in gastrointestinal malignancies. The acquisition of sustained proliferative signaling is one of the hallmark features of cancer, as recognized by cancer researchers (Hanahan and Weinberg, 2000). Therefore, the ability to inhibit tumor cell proliferation holds substantial importance in tumor therapy. However, it is important to note that other features are also crucial in the context of tumors. Gal-9 possesses multiple properties within the tumor immune microenvironment (TIME) and can be expressed to varying extents in nearly all immune cells (Chaudhary et al., 2022), including tumor-associated macrophages (TAM) (Qi et al., 2019), antigen-presenting cells (APCs) (Ashraf et al., 2017), Regulatory T-cells (Treg cells) (Ashraf et al., 2015), and T-cells.

Gal-9 plays a crucial role in tumor pathogenesis, and further research exploring its regulation in tumors can enhance our understanding of its mechanisms. Promising therapeutic activity against malignancies has been observed in relevant *in vivo* and *in vitro* experiments, as well as in preclinical models involving Gal-9. This review aimed to provide a comprehensive overview of changes in Gal-9 expression in hematologic malignancies and solid tumors, shedding light on their significance in these contexts. Furthermore, it delves into the intricate nature of Gal-9 as a multilateral partner in the TIME. It systematically elucidates Gal’s role in the TIME, establishing its status within this complex ecosystem. Moreover, this review proposes that targeting Gal-9 in combination with immunotherapy could present a new avenue for tumor treatment in the “post-immune era”, offering renewed hope in the field.

## 2 Overview of the structure and general biological functions of Galectin-9

Gal-9 belongs to the “tandem-repeat type” galectin family, which was originally recognized as a potent eosinophil chemotactic agent, and consists of an N-terminal Carbohydrate Recognition Domain (N-CRD) and a C-terminal CRD (C-CRD), each comprising 148 and 149 amino acids, respectively (Wiersma et al., 2013). The presence of the CRD is a defining structural hallmark shared by all members of the galectin family. Extensive studies by Nagae M et al. have explored the structural features of Gal-9, revealing that the overall structure of both the N-CRD and C-CRD is similar. The differences primarily arise from the insertion or deletion of amino acid residues, leading to specificity in carbohydrate binding (Nagae et al., 2006). The presence of N- and C-CRDs contributes to the biological effects of Gal-9, and studies have shown that N-CRDs activate dendritic cells more efficiently, whereas C-CRDs are major determinants of receptor recognition and death pathway signaling in T-cells (Wiersma et al., 2013).

Human Gal-9 has three natural isoforms: Gal-9 (S), Gal-9 (M), and Gal-9 (L), which differ in the length of the linker region separating the N-CRD and C-CRD. As mentioned earlier, alternative splicing of Gal-9 results in the selective expression of these isoforms in different cell types. For instance, T cells selectively overexpress Gal-9(M) and Gal-9(L) (Sato et al., 2002). In a review by Wiersma VR et al. (Wiersma et al., 2013), it was noted that the difference between Gal-9 isoforms is associated with the linkers and primarily affects the ability of the isoforms to bind to glucose ligands. However, this difference does not impact the function of Gal-9 in attracting eosinophils and inducing T cell apoptosis. Consequently, future investigations exploring the relationship between Gal-9 and tumor immune cells, as well as the development of immune drugs targeting Gal-9, may be less constrained by the isoform variations. Available data suggest that Gal-9 has a wide range of biological functions in normal cell biology and pathology. Under hyperimmune conditions, Gal-9 can act as a negative regulator of the immune system, while under immune compromised conditions, it can act as an immune enhancer. This role as a regulator of immune homeostasis makes it not surprising that Gal-9 its a candidate for the treatment of a wide range of diseases and gives it a place in the complex tumor immune microenvironment.

## 3 Role of Gal-9 in malignancy

**FIGURE 2:**
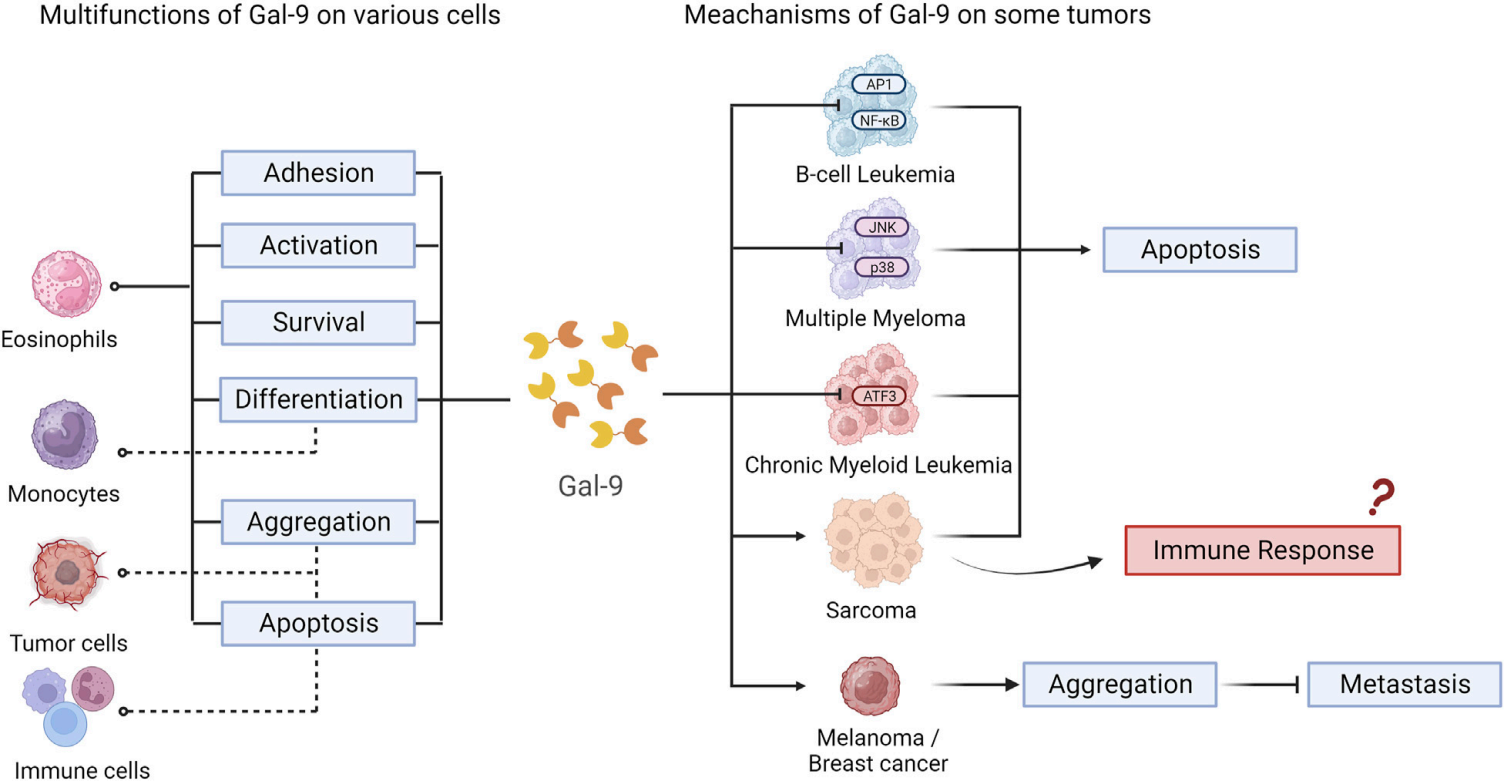
Multifunctions and meachanism of Gal-9 on various cells and tumors.

Gal-9 plays a role in various types of hematologic malignancies and solid tumors, as demonstrated in related cell-based studies and animal and clinical experiments (The data are shown in Table 1). In addition, common mechanisms of Gal-9 in multiple cell types and tumors were explored by us (Figure 2).

**TABLE 1:** Role of Gal-9 in various cancers.

Tumor type	Expression	Experiment type	References
AML	Gal-9 is upregulated in human AML cells	*In vitro*	Goncalves Silva et al. (2017)
	Gal-9 is upregulated in patients with who have failed chemotherapy	Review, Clinical trial	Dama et al. (2019)
	Gal-9 induces apoptosis of VISTA-expressing T cells	*In vitro*	Yasinska et al. (2020)
	High expression of Gal-9 is associated with a poor prognosis	*In vitro*, Clinical trial	Asayama et al. (2017)
CLL	Gal-9 is highly expressed in patients with CLL	Clinical trial	Pang et al. (2021)
	Gal-9 expression more pronounced in patients with advanced CLL than those in early stages	Clinical trial	Pang et al. (2021)
	High expression of Gal-9 promotes Treg cell proliferation and differentiation	Clinical trial	Pang et al. (2021)
	Inhibition of Gal-9 preserves CD26^+^CD8^+^ T cells	Review	Zheng et al. (2019)
ALL	Adipocytes-mediated Gal-9 can be upregulated in B-ALL	*In vitro*	Lee et al. (2022)
Malignant melanoma	Gal-9 can induce apoptosis in malignant melanoma cells	*In vitro*	Wiersma et al. (2012)
	Gal-9 is highly expressed in melanoma cell nevi and primary melanoma lesions	*In vitro*	Kageshita et al. (2002)
	Gal-9 is not or rarely expressed in metastatic melanoma lesions	*In vitro*	Kageshita et al. (2002)
	Ectopic expression of Gal-9 can inhibit Gal-9-deficient melanoma cell metastasis	*In vivo*	Nobumoto et al. (2008)
	The expression of Gal-9 in melanoma is positively correlated with an increase in the number of CD206+ macrophages, promoting tumor growth	*In vitro*	Enninga et al. (2018)
	Gal-9+ and PD-L1+ were co-expressed in the CCR + melanoma cell subpopulation to promote tumor metastasis	*In vitro*, Clinical trial	Cristiani et al. (2019)
	Gal-9+ dendritic cells/dendritic cell-like macrophages are parameters for higher survival rates in advanced melanoma	Clinical trial	Melief et al. (2017)
Cervical cancer	Gal-9 is highly expressed in the nucleus and cytoplasm of cervical cancer cells	Clinical trial	Curley et al. (2020)
	Gal-9 is expressed in all immune cells of cervical cancer cells and in aggressive and intraepithelial tumors	Clinical trial	Curley et al. (2020)
Colon cancer	Exogenous rLGALS9 triggers the death of colorectal cancer cells with refractory KRAS gene mutations by mediating clathrin- and PRKC (protein kinase C)-, RAF1-, and MAP2K1-	*In vitro*, *In vivo*	Wiersma et al. (2015)
	Gal-9 expression is elevated in colorectal cancer	*In vitro*, Clinical trial	Sasidharan Nair et al. (2018), Blair et al. (2021)
	Gal-9 can inhibit the proliferation of human colon cancer cells by inducing apoptosis	*In vitro*, *In vivo*	Morishita et al. (2021)
	The upregulation of Gal-9 in colorectal cancer can lead to its immune evasion	*In vitro*	Sasidharan Nair et al. (2018)
Breast cancer	Gal-9 on the surface of breast cancer cells can be translocated by FLRT3/LPHN/TIM-3/Gal-9	*In vitro*	Yasinska et al. (2019)
	The high expression of Gal-9 in breast cancer can inhibit breast cancer metastasis	*In vitro*, Clinical trial	Irie et al. (2005), Yamauchi et al. (2006)
	High expression of Gal-9 in breast cancer epithelial cells enhances the early aggressiveness of breast cancer	*In vitro*, Clinical trial	Pally et al. (2022)
	The expression of Gal-9 and TIM-3 is associated with a favorable prognosis for triple-negative breast cancer	Clinical trial	Yoshikawa et al. (2022)
	Gal-9 is upregulated under cytotoxic drug intervention in triple-negative breast cancer	*In vitro*	Yoon et al. (2018)
Lung cancer	Gal-9 is expressed on all non-small cell lung cancer cells and tumor-infiltrating lymphocytes	Clinical trial	He et al. (2019)
	The expression of Gal-9 is elevated in lung cancer, and the expression level is significantly different from that of the healthy group	Clinical trial	Blair et al. (2021)
	Gal-9 is expressed more in stage I and IV lung cancer than in stage II and III	Clinical trial	
	Gal-9 expression is more frequent in women than in men	Clinical trial	
	Gal-9 expression higher in patients who are smokers than in non-smokers	Clinical trial	
Hepatocarcinoma	Patients with high expression of Gal-9 in hepatocellular carcinoma have a better prognosis	Clinical trial	Gu et al. (2013)
	Gal-9 in hepatocellular carcinoma has different levels of expression in antigen-presenting cell subsets	*In vitro*, Clinical trial	Li et al. (2012)
	The downregulation of Gal-9 within hepatocellular carcinoma cells is associated with tumor growth, tumor migration, invasion, metastasis, postoperative recurrence, and poor prognosis	Review	Bacigalupo et al. (2013)
	Low expression of Gal-9 is associated with a poorer prognosis in patients with hepatocellular carcinoma	Clinical trial	An et al. (2021)
Gastric cancer	The expression of Gal-9 in patients with gastric cancer is associated with clinical stage, tumor pathological stage, tumor cell differentiation, lymph node metastasis, and survival	Clinical trial	Yang et al. (2014)
	Gal-9 is highly expressed in gastric cancer cells	Clinical trial	Wang et al. (2018)
	The expression of Gal-9 and TIM-3 negatively correlated with the overall survival rate of patients	Clinical trial	Wang et al. (2018)
	Gal-9 inhibits gastric cancer cell invasion, migration, and epithelial-mesenchymal transformation under the regulation of PPARγ	*In vitro*, *In vivo*	Cho et al. (2015)
	WEE1 inhibitors in HER2-positive gastric cancer downregulated Gal-9 expression to improve trastuzumab resistance	*In vitro*, *In vivo*	Jin et al. (2021)
	Low expression of Gal-9 is significantly associated with the prognosis of gastric cancer patients	Review	Long et al. (2018)
Glioblastoma	Gal-9 is highly expressed in the brain tissue of glioma patients	Clinical trial	Yuan et al. (2020)
	High expression of Gal-9 negatively correlated with overall survival	Clinical trial	Yuan et al. (2020)

### 3.1 Hematologic malignancy

#### 3.1.1 Acute myeloid leukemia (AML) and myelodysplastic syndromes (MDS)

A study conducted by Kikushige Y et al. (Kikushige et al., 2015) in 2015 reported that human AML cells can secrete large amounts of Gal-9 into the bloodstream. Gal-9 expression is upregulated in patients with AML who had an unsuccessful chemotherapy (Dama et al., 2019). The study showed that Gal-9 in AML cells can bind to its receptor T cell immunoglobulin and mucin-domain containing-3 (TIM-3), which is expressed on the surface of AML stem cells. Reports also suggest that the interaction between Gal-9 and TIM-3 supports the maintenance of leukemia-initiating cells and promotes immune eversion in AML models (Goncalves Silva et al., 2017; Ruvolo, 2019; Gallazzi et al., 2022). Yasinska IM et al. (Yasinska et al., 2020) reported that Gal-9 produced by AML cells can induce a pro-apoptotic process in T cells expressing V-domain Ig-containing suppressors of T cell activation (VISTA). However, Gal-9 does not promote apoptosis in natural killer cells (NK cells) that do not express VISTA. The VISTA protein expressed by T cells recognizes Gal-9 secreted by AML cells and acts as its ligand. Simultaneously, soluble VISTA released by AML cells enhances the action of Gal-9, leading to the formation of a multiprotein complex on the surface of T cells. This complex acts as a molecular barrier, ultimately activating granzyme B in cytotoxic T cells and triggering apoptosis. Clinical data suggests that patients with AML exhibit elevated levels of plasma Gal-9, and the levels are associated with a poor prognosis for the disease (Asayama et al., 2017). Additionally, approximately 10%–40% of patients with MDS progress to AML. Studies have demonstrated significantly higher levels of plasma Gal-9 in patients with MDS and those progressing from MDS to the AML stage compared to normal individuals.

#### 3.1.2 Chronic lymphocytic leukemia (CLL)

Previous studies have demonstrated that Gal-9 expression is elevated in peripheral blood cells of patients with CLL, particularly in those with advanced stages of the disease (Taghiloo et al., 2017). This indicates a potential association between Gal-9 expression and disease progression in CLL. Pang et al. (2021) further confirmed the elevation of Gal-9/TIM-3 in CLL and its correlation with disease progression. Serum levels of Gal-9 and IL-10 are elevated in patients with CLL, particularly in patients in Binet stages B and C, whereas the levels of interferon-γ (IFN-γ) are decreased. Based on these findings, Zheng et al. (2019) raised the possibility that elevated Gal-9 levels in CLL may contribute to immune dysfunction characterized by the aggregation of non-functional and exhausted CD8^+^ T cells. The immune system plays a critical role in inhibiting tumor cell proliferation (Gajewski et al., 2013), and the impairment of the immune system mediated by Gal-9 may potentially promote the proliferation of leukemia cells in CLL.

#### 3.1.3 B-cell acute lymphoblastic leukemia (B-ALL)

Acute lymphoblastic leukemia (ALL) is a malignant disease characterized by the abnormal proliferation of B- or T-lineage cells in the bone marrow. CAR-T cell therapy has been used for the treatment of acute B-lymphocytic leukemia (BLL) for over a decade, showing clinical benefit. However, the emergence of additional data has identified patient-specific risk factors associated with this therapy. The influence of obesity factors in B-ALL has been investigated, and a study revealed that adipocyte secretomes can upregulate the expression of Gal-9 on the surface of human acute B-ALL cells, leading to an increased chemoresistance (Lee et al., 2022). The study also demonstrated that the upregulation of Gal-9 on B-ALL cells mediated by adipocytes could potentially overcome obesity-induced chemoresistance through antibody-based therapy.

Gal-9 signaling in AML cells activates the AKT and ERK signaling pathways. Interestingly, exposure of B-ALL cells to factors secreted by adipocytes also leads to activation of these signaling pathways. This suggests a potential shared mechanism involving Gal-9 in different leukemia subtypes. Studies are currently underway to explore the early integration of immunotherapeutic agents such as inotuzumab and blinatumomab with low-dose chemotherapy (dose-dense mini-Hyper-CVD-inotuzumab-blinatumomab) in the frontline setting for the treatment of B-ALL (Jabbour et al., 2023). Following this approach, CAR T-cell consolidation at high doses without maintenance therapy is being proposed for at-risk patients Inhibition of Gal-9 represents an unexplored approach that could potentially improve the prognosis of patients with B-ALL and other leukemia subtypes, particularly in the presence of obesity-related factors.

### 3.2 Solid malignancies

#### 3.2.1 Malignant melanoma

The existing data suggest that Gal-9 plays a tumor-suppressive role in malignant melanoma (Wiersma et al., 2012). Studies have shown that the absence of Gal-9 is strongly associated with metastatic progression and that recombinant Gal-9 has hitherto unrecognized cytotoxic effects on human melanoma cells. Previous studies have reported that Gal-9 expression is high in nevi and primary melanoma lesions but low in metastatic melanoma lesions (Kageshita et al., 2002). Ectopic expression of Gal-9 inhibits metastasis in Gal-9-deficient B16F10 mouse melanoma cells (Nobumoto et al., 2008). And Gal-9, through its interaction with CD206 on M2 macrophages, promotes angiogenesis, chemokine production, and tumor growth, with corresponding poor patient prognosis (Enninga et al., 2018). However, several recent studies have shown that Gal-9 can be involved in melanoma metastasis under specific conditions, and CCR7 is highly expressed in metastases from melanoma patients, where a subpopulation of CCR7+ melanoma cells was found to co-express PD-L1 and Gal-9 (Cristiani et al., 2019). A study by Melief SM et al. identified the presence of Gal-9+ dendritic cells/dendritic cell-like macrophages to be associated with better survival in patients with stage IV melanoma (Melief et al., 2017).

In addition, with the advent of targeted therapies and checkpoint inhibitors, adoptive cell transfer has become an important salvage therapy for patients with stage IV melanoma. The emergence of Gal-9+ dendritic cells/dendritic cell-like macrophages is most commonly seen in patients with sustained clinical benefit following adoptive cell transfer therapy. Epigenetic regulation of TIM-3 and Gal-9 in malignant melanoma has been innovatively explored by Holderried et al. (2019), specifically through DNA methylation. Although studies on Gal-9 in melanoma are scattered and it is not yet possible to summarize the common mechanisms, the above studies provide insights into Gal-9 as a predictive bioinformatic marker in the future.

#### 3.2.2 Colon cancer

Exogenous rLGALS9, a lysosome inhibitor, exhibits high cytotoxicity against KRAS-mutated colorectal cancer cells (Wiersma et al., 2015). Exogenous rLGALS9 can undergo rapid internalization through clathrin- and protein kinase C-, RAF1-, and MAP2K1-dependent endocytosis, resulting in the accumulation of rLGALS9 in lysosomes. This process triggers cell death in colorectal cancer cells harboring refractory KRAS gene mutations. Blair et al. (2021) conducted an ELISA-based comparison of galactose lectin concentrations across different stages of multiple cancer types and found a statistically significant increase in Gal-9 levels in colon cancer. The impact of Gal-9 on human colorectal cancer cells has been directly demonstrated in studies by Morishita et al. (2021) and Sasidharan Nair et al. (2018) They showed that Gal-9 can inhibit cell proliferation through apoptosis in both *in vivo* and *in vitro* experiments. Sasidharan Nair et al. (2018) focused on exploring the influence of the Gal-9 promoter on colorectal cancer through epigenetic modifications such as DNA methylation. Notably, a meta-analysis showed that high expression of Gal-9 had a positive impact on overall survival (OS) in patients with colorectal cancer (Zhou et al., 2018). High Gal-9 expression was associated with improved OS, but did not correlate with disease-free survival (DFS) or recurrence-free survival (RFS) in solid tumors. In conclusion, the positive impact of high Gal-9 expression in colon cancer is a consensus topic in both clinical and *in vitro* experimental studies.

#### 3.2.3 Breast cancer

Gal-9 on the surface of breast cancer cells acts as a protective agent against cytotoxic T-cell-induced cell death. A series of studies by Yamauchi A et al. found that Gal-9 appears to protect Gal-9-expressing tumors from immune attack, and this protective effect does stop breast cancer from metastasizing (Irie et al., 2005; Yamauchi et al., 2006; Gleason et al., 2012). Pally et al. (2022) demonstrated the high expression of Gal-9 in breast cancer epithelial cells through *in vitro* cell experiments, clinical samples, and analysis of publicly available database samples. The authors also revealed that high Gal-9 expression enhances the invasiveness of breast cancer cells in the early stages of invasion. Contrary to the findings of previous studies, there was a positive correlation between Gal-9 levels and the invasive potential of cancer cells, suggesting a potential role for Gal-9 in inducing breast cancer cell invasion, and posing a challenge for the potential development of Gal-9-targeted inhibitors in breast cancer. With the development of spatial biology and spatial histology, the paradox of Gal-9 in breast cancer may be resolved in the future, and spatial variability among single cells may answer why there is variability in Gal-9 findings in breast cancer. Despite the uncertainties regarding the role of Gal-9, the presence of both Gal-9 and TIM-3 in very heterogeneous triple-negative breast cancer (TNBC) has been significantly associated with a favorable prognosis (Yoshikawa et al., 2022).

As an immune checkpoint protein, Gal-9 has received less attention in the immune microenvironment of TNBC. Liu C. et al. (2022) identified multiple tumor immune risk score groups for TNBC, which established different types of immune microenvironments based on immune molecular markers. This research provides new insights into immunotherapy for TNBC. Previously, Ju et al. (2021) examined the relationship between Gal-9 and clinicopathological features, tumor-infiltrating lymphocyte (TIL) levels, PD-L1+ immune cells, and tumor cells in TNBC. The results suggested that increased Gal-9 expression is associated with higher immune cell infiltration and beneficial changes in terms of metastasis and other factors. Additionally, anthracyclines and taxanes are chemotherapeutic agents widely used in the adjuvant and neoadjuvant treatment of early breast cancer (Zaheed et al., 2019), Yoon et al. (2018) found a potential association between changes in PD-L1 and Gal-9 expression in TNBC and chemotherapy (anthracyclines and taxanes). A recent study showed that the anthracyclines doxorubicin and epirubicin induced Gal-9 expression via the STING/IF axis, and that the combination of chemotherapy and anti-Gal-9 produced synergistic antitumor effects (Sun et al., 2023). Although these studies did not extensively cover the drug selection process, it demonstrated the impact of cytotoxic drugs on Gal-9 expression in TNBC, providing a basis for future clinical trials evaluating combination therapies involving PD-L1 and Gal-9.

#### 3.2.4 Lung cancer

The investigation of Gal-9 in lung cancer encompasses various types of lung cancer, including non-small cell lung cancer (NSCLC) (He et al., 2019), small cell lung cancer (SCLC) (Chen et al., 2020), lung large cell neuroendocrine carcinoma (LCNEC) (Che et al., 2022), and even the rare pulmonary sarcomatoid carcinoma (PSC) (Guo et al., 2021).

Chen et al. (2020) and Che et al. (2022) developed Gal-9-based immune risk score models to predict the recurrence prognosis of SCLC and LCNEC, respectively. Both studies, conducted in different lung cancer types, showed a significant association between Gal-9 and TIME and immune infiltration in lung cancer, particularly in stage I-III SCLC and LCNEC, where Gal-9 exhibited unique prognostic value. He et al. (2019) identified Gal-9 expression on NSCLC cells and TIL through immunohistochemistry. The expression of Gal-9 on tumor cells showed a significant correlation with survival, while Gal-9 expression on TILs was strongly associated with early postoperative recurrence. Blair et al. (2021) reported elevated Gal-9 expression in lung cancer compared to healthy controls, with differential expression observed at different stages of the disease among patients of different sexes and daily habits. Unlike other cancers, Gal-9 research in lung cancer has focused more on clinical studies, and how Gal-9 actually affects the development of lung cancer is still enigmatic in the current stage of research, and it is an urgent issue to dig out the effect of Gal-9 in lung cancer from the tissue or even single-cell level in the future.

#### 3.2.5 Hepatocellular carcinoma (HCC)

Previous studies have demonstrated that Gal-9 is a prognostic factor for HCC with anti-metastatic potential (Gu et al., 2013). Patients with high Gal-9 expression in HCC tend to have a better prognosis. Zhang et al. (2012) utilized the Cox proportional hazard model, which indicated that negative Gal-9 expression in HCC represents a potential risk factor for patient survival.

Chronic hepatitis B virus (HBV) infection is a major risk factor for HCC (Bray et al., 2018), and identifying prognostic biomarkers for HBV-associated HCC is crucial (Mani and Andrisani, 2018). In HBV-associated HCC, Jiao et al. (2022) conducted univariate and multivariate analyses, revealing that Gal-9 could serve as an independent prognostic marker. Li et al. (2012) detected varying levels of Gal-9 expression in APC subpopulations in HCC, highlighting the role of the Gal-9-related signaling pathway in T-cell senescence in HBV-associated HCC. Furthermore, Gal-9 downregulation in HCC cells has been associated with tumor growth, migration and invasion, metastasis, postoperative recurrence, and a poor prognosis (Bacigalupo et al., 2013). A recent systemic evaluation and meta-analysis also linked low Gal-9 expression with a worse prognosis in patients with HCC (An et al., 2021). Galactose lectins have demonstrated involvement in other liver pathologies characterized by chronic inflammation and/or fibrosis (Okwor et al., 2020). While galactose lectin-based therapies have been proposed for the treatment of liver lesions like HCC, functional studies are needed to elucidate the precise molecular mechanisms by which galactose lectins contribute to HCC. Notably, although numerous studies have explored the role of Gal-9 in hepatocellular carcinoma, Kong F et al. concluded that Gal-3 outperforms Gal-9 as a novel prognostic marker in HCC, suggesting that Gal-9 expression is primarily associated with tumor progression (Kong et al., 2020).

#### 3.2.6 Gastric cancer (GC)

In 2014, a pioneering study explored the role of Gal-9 in clinically diagnosed primary GC tissue (Yang et al., 2014). The study examined the correlation between Gal-9 expression and a variety of clinical factors in GC patients. However, no significant association was found between Gal-9 expression and distant metastasis (*p* > 0.05). In this study (Wang et al., 2018), more than two-fold reduction in TIM-3 expression was observed in 59% of GC tissues compared to normal tissues, which aligns with the findings of Wang Y et al. Nevertheless, no correlation between Gal-9 and TIM-3 was identified in GC. Another significant factor investigated in this context is peroxisome proliferator-activated receptor γ (PPARγ), which possesses anti-proliferative and pro-differentiation effects in various cancer types (Girnun et al., 2002; Elrod and Sun, 2008; Tontonoz and Spiegelman, 2008).

In both *in vitro* experiments and a systematic evaluation and meta-analysis, Gal-9 has emerged as a promising therapeutic target for the treatment of GC (Takano et al., 2016; Zhou et al., 2018). In an *in vitro* study conducted by Takano et al. (2016), it was visually demonstrated that Gal-9 induced apoptosis in GC by modulating miRNAs, leading to the inhibition of cancer cell proliferation. Expanding on Gal-9’s role in GC, Jin et al. (2021). Employed Gal-9 as a strategy to enhance resistance to trastuzumab in HER2-positive GC. Their findings revealed that simultaneous targeting of HER2 and WEE1 could overcome trastuzumab resistance in HER2-positive GC. Trastuzumab, known to upregulate PD-L1 via NF-kB activation (Chaganty et al., 2018; Su et al., 2018), can be counteracted by WEE1 inhibitors, which downregulate PD-L1 expression while reducing levels of Gal-9, CD163, and CTLA-4. This approach improves resistance to trastuzumab in HER2+ GC.

Additionally, a retrospective case-control study from 2018 of 2098 patients with GC revealed a noteworthy connection between low Gal-9 expression and patient prognosis (Long et al., 2018). The expression level of Gal-9 is associated with clinical features and has been acknowledged as a potential independent prognostic predictor for patients with GC (Wang R. et al., 2020).

#### 3.2.7 Glioblastoma (GB)

In GB, Gal-9 is expressed along with multiple family partners, such as Gal-1, Gal-3, and Gal-8. The interaction of Gal-9 with their respective sugar-carrying molecules hinders the antitumor response, thereby promoting immunosuppression. While Gal-9’s immunosuppressive mechanism in GB is closely linked to TIM-3, it also plays an important role independently within GB.

Yuan et al. (2020) conducted a comprehensive analysis of 1,027 patients with glioma and found a strong upregulation of Gal-9 in GB compared to normal brain tissue. Moreover, high levels of Gal-9 expression were associated with a shorter OS in patients with low-grade gliomas. In GB tissue samples, Gal-9 expression was correlated with the presence of immunosuppressive M2-macrophages, which showed a positive correlation with immune checkpoint molecules (Knudsen et al., 2021).

#### 3.2.8 Other solid tumors

Apart from the solid tumors mentioned earlier, Gal-9 also plays a role in cervical and vulvar aquamous cell carcinomas, esophageal adenocarcinoma, esophageal squamous cell carcinoma, gallbladder carcinoma, and bile duct carcinoma. However, clinical research on these tumors is limited. Gal-9 exhibits distinct staining patterns in cervical and vulvar squamous cell carcinomas. While nuclear/cytoplasmic staining is typically reduced in the presence of membrane staining, both tumor types show strong expression in the nucleus and cytoplasm. This distribution pattern may be linked to the broader activity range of the Gals family (Curley et al., 2020). In esophageal adenocarcinoma (EAC) (Akashi et al., 2017), Gal-9 regulates proliferation by inducing apoptosis, autophagy, and affecting angiogenesis. It inhibits the proliferation of EAC cell lines such as OE19, OE33, SK-GT4, and OACM 5.1C, increases the expression of CCK18 and IL-8, and activates caspase-3 and caspase-9. Moreover, in esophageal squamous cell carcinoma (ESCC) (Akashi et al., 2017), Gal-9 inhibits cell proliferation through concentration-dependent activation of JNK and p38 and induces mitochondria-mediated apoptosis in ESCC cells. In gallbladder and cholangiocarcinomas (Kobayashi et al., 2015; Tadokoro et al., 2016), Gal-9 inhibits the proliferation of various cancer cells. However, in gallbladder cancer, it does not inhibit the OCUG-1 cell line, which represents a hypodifferentiated type of adenosquamous carcinoma. In both gallbladder and cholangiocarcinomas, Gal-9 induces apoptosis through different pathways. In gallbladder cancer, it increases the levels of CCK18 and phosphorylated p53. In cholangiocarcinoma, Gal-9 induces apoptosis through an intrinsic apoptotic pathway mediated by cysteinase-dependent or non-independent pathways. It decreases the phosphorylation of various growth factor receptors and increases the expression of CCK18 and cytochrome c in cholangiocarcinoma cell lines. These findings highlight the role of Gal-9 in gastrointestinal malignancies. Concurrent with the previous studies on other gastrointestinal malignancies such as gastric cancer and colorectal cancer, it becomes evident that Gal-9 is involved in the “unlimited proliferation” and “apoptosis” characteristics of these tumors.

## 4 Multilateral partner of TIME

The significant impact of Gal-9 on various malignant hematological diseases and solid tumors has been discussed and summarized earlier. It plays a crucial role as an immune checkpoint in multiple cancer sites, affecting various functions of tumor cells. This discussion aimed to highlight Gal-9 as an “independent entity” in the immune microenvironment.

**FIGURE 3:**
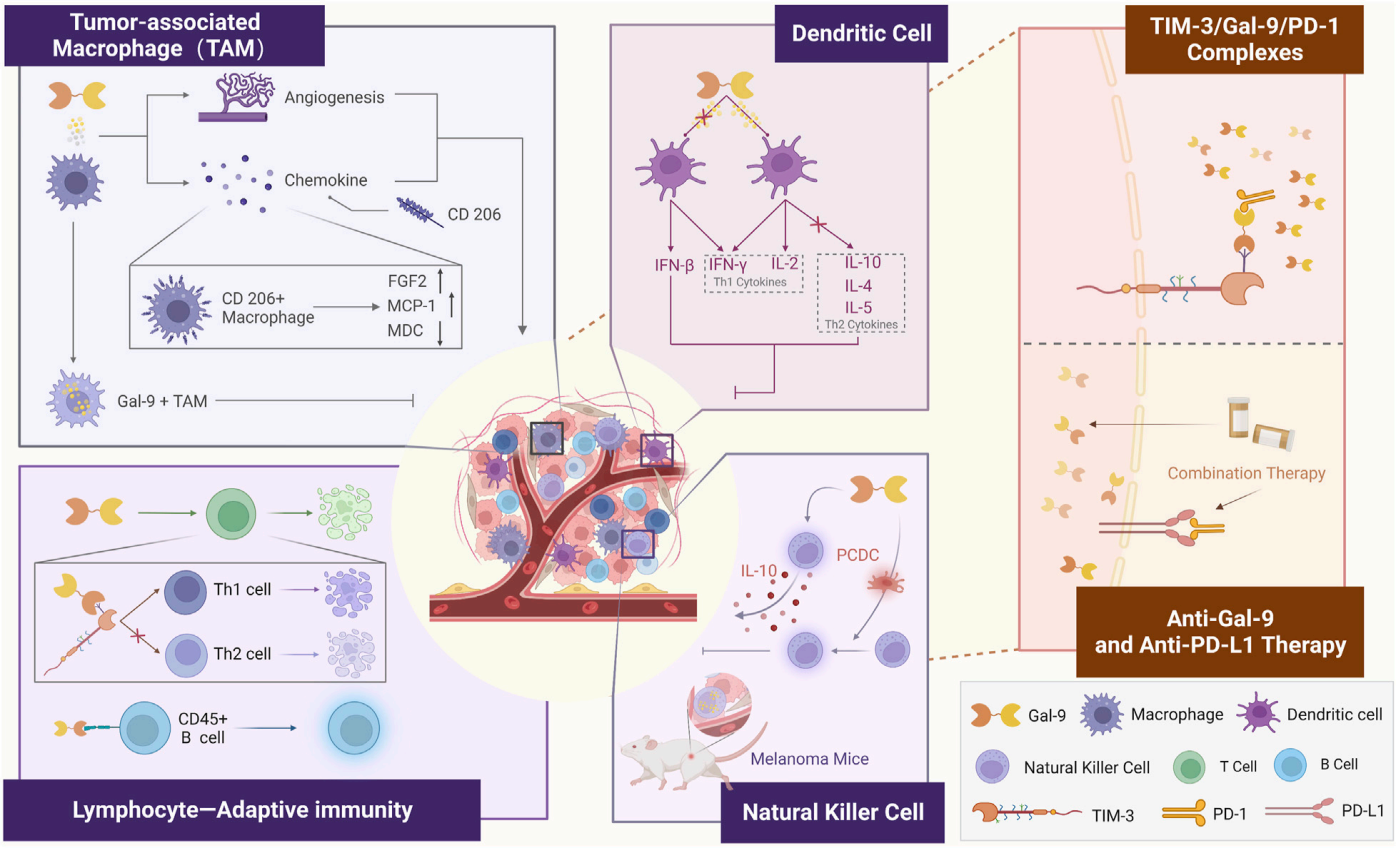
Role of Gal-9 as a “Multilateral Partner” in the tumor immune microenvironment.

Gal-9 has been identified as a “negative regulator of the adaptive immune response”, primarily through its binding to TIM-3, which is widely expressed in immune cells, particularly T-cells (Cedeno-Laurent and Dimitroff, 2012). The TIME is a highly complex ecosystem in which the human immune system plays a role in inhibiting tumor cell proliferation and promoting tumor cell apoptosis (Gajewski et al., 2013). Gal-9 exhibits interactions with multiple immune cells. Studies have shown that Gal-9 enhances anti-tumor immunity in mice with tumors by promoting the maturation of CD11c+ DCs, which in turn activate CD4^+^ TIM-3+ and CD8^+^ TIM-3+ T cells. In addition, Gal-9 activates plasmacytoid dendritic cell-like macrophages (PCDC), leading to the activation of NK cells. This highlights Gal-9’s role as a “Multilateral Partner” within the TIME (The figure is depicted in Figure 3).

### 4.1 Macrophages

Macrophages play a dual role within the TIME, and TAMs are a crucial component of immune cells in the TIME. A report demonstrated that Gal-9+ TAM predicts OS, RFS, and response to adjuvant chemotherapy in patients with muscle-invasive bladder cancer (MIBC) (Qi et al., 2019). The expression of Gal-9 in bone marrow-derived macrophages (BMDM) is influenced by the time-dependent effects of lipopolysaccharide (LPS) simulation. Short-term LPS increases Gal-9 expression and secretion, activating the TIM-3/Gal-9 signaling pathway, which inhibits M1 polarization. On the other hand, long-term stimulation reduces Gal-9 expression and secretion, activating the TIM-3 and ultimately promoting M1 polarization (Kobayashi et al., 2015). In a report on GB, Gal-9 was highly associated with immune checkpoint molecules and M2 tumor-associated macrophages (Yuan et al., 2020). Recent research on oncogene-deficient phosphatase and tensin homolog (PTEN) GB, revealed that basement membrane cells deficient in PTEN secrete significant amounts of Gal-9 via the AKT-GSK3β-IRF1 pathway (Yuan et al., 2020). This Gal-9 secretion drives M2 polarization in macrophages by activating the TIM-3 receptor and downstream pathways. In melanoma-bearing mice, Gal-9 increases the number of macrophages, whereas Gal-9-mediated antitumor activity was not induced in macrophage-deficient mice (Nobumoto et al., 2009). Gal-9 also promotes NK cell-mediated antitumor activity by amplifying a distinct subset of macrophages that exhibit a plasma cell-like phenotype. Additionally, Gal-9 interacts with macrophages in patients with metastatic melanoma, causing CD206+ macrophages to produce increased levels of FGF2 and monocyte chemotactic protein-1 (MCP-1) while reducing macrophage-derived chemokines (MDC) (Enninga et al., 2018). This suggests that Gal-9 plays a role in promoting the production of angiogenic chemokines in malignant melanoma through its interaction with macrophages. Although Gal-9 polarizes macrophages differently depending on the available conditions, we believe that the role of Gal-9 and macrophages within this environment is consistent. TAMs with high expression of Gal-9 inhibit tumor cell activity in the absence of intervention by other factors.

### 4.2 NK cells

Differences in the effector function of Gal-9+ NK cells between mice and humans should be considered in different physiological and pathological conditions. A recent study revealed the expansion of Gal-9+ NK cells in the tumor tissue of melanoma mice (Rahmati et al., 2023). The authors of the study, Meggyes et al. (2021), demonstrated that Gal-9 intervention led to the production of IL-10 by NK-92MI cells. IL-10 is a potent immunosuppressive cytokine that facilitates tumor cell evasion from APCs and impairs helper T cell-mediated immune surveillance. Consequently, this suggests that the cytotoxic effect of NK cells is reduced primarily through secondary mechanisms (D'Andrea et al., 1993). This finding has inspired further investigation to determine the precise function induced by the increased IL-10 levels following Gal-9 treatment.

### 4.3 Dendritic cells

Alterations in the activity and phenotype of dendritic cells play a role in the progression of various diseases. For instance, in patients with endometriosis, the accumulation of myeloid DCs expressing Gal-9 and plasmacytoid DCs in the peritoneal fluid is considered a characteristic feature of immune regulation (Suszczyk et al., 2023). Similarly, in GB, Gal-9 serves as a marker of immune regulation: After Gal-9 intervention, do not secrete IL-10 but instead produce IL-2 (Liberal et al., 2012). Gal-9 induces the maturation of DCs and promotes Th1 immune responses in adaptive immunity (Liberal et al., 2012). DCs can generate IFN-β, and the combined effect of IFN-β and IFN-γ increases the expression of Gal-9 in APCs, including B cells, DCs, and TAMs. This upregulation of Gal-9 inhibits anti-tumor responses by inducing T cell death (Yang et al., 2021).

### 4.4 Lymphocytes

In the context of adaptive immunity, CD4^+^ T cells differentiate into two subsets, namely, helper T cells 1 (Th1 cells) and helper T cells 2 (Th2 cells), in response to different antigenic stimuli (Mosmann and Coffman, 1989). Both CD4^+^ and CD8^+^ T cells are sensitive to Gal-9-induced cell death, but CD8^+^ cytotoxic T cells are considered more sensitive than CD4^+^ T cells (Yang et al., 2021). Th1 cells produce IL-2 and IFN-γ, which induce a delayed hypersensitivity response and promote immunity (Paul and Seder, 1994). On the other hand, Th2 cells produce IL-4, IL-5, IL-10, and IL-13, which are involved in immunoglobulin G1 (IgG1) and IgE production as well as eosinophilic inflammation (Paul and Seder, 1994). The cytokines produced by Th1 and Th2 cells have opposing roles in immune regulation and can cross-regulate and exhibit mutual functional inhibition (Street and Mosmann, 1991). However, dysregulation of Th1 and Th2 responses can lead to pathological consequences. Overactive Th1 responses may cause organ-specific autoimmune diseases such as type I diabetes and multiple sclerosis (Romagnani, 1994; Kamradt and Mitchison, 2001), while uncontrolled Th2 responses are associated with allergies and sensitivities (Sher and Coffman, 1992; Abbas et al., 1996).

Functional studies have demonstrated that Gal-9 induces Th1 cell death via TIM-3, but not Th2 cell death (Zhu et al., 2005). In immunized mice, the injection of Gal-9 substantially reduces the number of IFN-γ-producing Th1 cells (Zhu et al., 2005). In studies related to AML (Kashio et al., 2003), AML cells impaired the anticancer activity of cytotoxic lymphocytes, including NK cells and T lymphocytes. The mechanism of T-cell death induced by Gal-9 has been investigated, particularly in leukemic T-cell lines. High doses of Gal-9 induce apoptosis in various T-cell leukemia cell lines in a dose-dependent manner, depending on the presence of CRDs in their structure. Interestingly, T cells themselves express and release the Gal-9 isoforms Gal-9 (M) and Gal-9 (L), and the sensitivity of T cells to Gal-9 is negatively correlated with their expression of Gal-9 (Hirashima, 2000; Chabot et al., 2002). Gal-9 induces T cell death in a variety of ways (Wiersma et al., 2013), including caspase-dependent apoptosis, Ca^2+^/Calpain activation, release of pro-apoptotic mitochondrial factors, or inhibition of cell cycle progression. In 2018, Giovannone et al. (2018) demonstrated the role of Gal-9 as an intrinsic B cell regulator of B cell receptor signaling and activation and that naive and memory B cells strongly bind Gal-9, and naive B cells express Gal-9 in blood and lymphoid tissue. Notably, CD45 was identified as the primary receptor for Gal-9 on naïve B cells, and binding of Gal-9 to CD45 activates inhibitory signaling pathways via the Lyn-CD22-SHP-1 pathway. Gal-9 functions as an inhibitor of B-cell receptor signaling, and its downregulation may contribute to a lower activation threshold of memory B cells.

## 5 Prospect of Gal-9 in the tumor immuno therapy

### 5.1 Gal-9 and its ligand TIM-3: the future of tumor immunotherapy

Gals are considered largely as modulators of the tumor immune response (Yang et al., 2021). There is growing interest in the future development of effective methods to modulate the effects of Gal-9 on tumors and autoimmunity. Previously, we identified co-regulatory pathways associated with Gal-9 in certain tumors, such as Gal-9/TIM-3. The Gal-9/TIM-3 pathway was described as a critical stimulatory loop for leukemic stem cells by Kikushige et al. This loop leads to Th1-mediated immune dysfunction and T cell apoptosis.

The dysregulation of immune responses plays a crucial role in the development of AML/MDS, opening up new possibilities for immunotherapy (Gallazzi et al., 2022). Sabatolimab (MBG453), although an inhibitor of TIM-3, disrupts the interaction between TIM-3 and Gal-9, showing promising results in the treatment of AML/MDS. A study utilizing multi-parameter flow cytometry analyzed peripheral blood and bone marrow biopsy specimens from 26 patients with AML at the time of diagnosis and after induction therapy (Dama et al., 2019). The study found that targeting the Gal9/TIM-3 pathway in combination with induction chemotherapy could enhance the likelihood of achieving complete remission in AML.

Earlier studies have shown that Gal-9 binds to TIM-3 and induces apoptosis by inducing TH1 cell death via intracellular calcium flow. This would therefore lead to induction of immune tolerance and suppression of TH1 and TH17 responses (Zhu et al., 2005). In breast cancer, where breast cancer cells do not secrete Gal-9, a study by Yasinska et al. (2019) confirmed the higher expression levels of Gal-9 and TIM-3 were found in breast cancer tissues compared to healthy breast tissues, and activation of the FLRT3/LPHN/TIM-3/Gal-9 pathway specifically promoted the translocation of Gal-9 on the surface of the tumor cell surface.

Notably, Gal-9 can exert its function through the release of extracellular vesicles. A noteworthy study demonstrated that Gal-9-enriched exosomes derived from GB impair the function of dendritic cells (DCs) and CD8^+^ T cells by binding to TIM-3, thereby facilitating tumor progression (Wang M. et al., 2020). The activity of exosomal Gal-9 on DCs is dependent on TIM-3, and knocking down TIM-3 in DCs restores their function and activation. As a result, it has been suggested as a potential therapeutic target to disrupt cell-cell interactions and exosome communication.

On the other hand, despite efforts to address the limitations of PD-1/PD-L1 immunotherapy with plant-derived compounds, drug resistance remains a challenging issue in current clinical oncology treatment (Liu K. et al., 2022). In a study by Yang et al. (2021), it was found that PD-1 can bind to Gal-9 on C-CRD, forming a competitive relationship with Gal-9/TIM-3. The formation of TIM-3/Gal-9/PD-1 complexes contributes to the persistence of PD-1+ TIM-3+ T cells and weakens Gal-9/TIM-3-induced cell death. These findings shed light on the intricate battle between cancer cells and the immune system. Based on these key observations and the significant anti-cancer activity of anti-Gal-9 antibodies, Gal-9 has become an attractive target for cancer therapy and Gal-9, either alone or in combination with TIM-3, plays a crucial role in maintaining immunosuppression within the tumor microenvironment.

### 5.2 Gal-9-related immune combination therapies: new directions in tumor immunotherapy

The diverse range of chemotherapy drugs can effectively enhance the efficacy and response of cancer patients, indicating that the combination of multiple immune checkpoints may address the limitations of single immune checkpoint inhibitors (Liu J. et al., 2022). Research suggests that Gal-9 is actually a protein that facilitates the escape of cancer from immune surveillance (Wiersma et al., 2015). The study by Yang, R., et al. evaluated the potential of Gal-9 inhibition in tumor therapy from a T cell perspective, where the preferential killing of T cells over cancer cells by anti-Gal-9 antibodies might contribute to tumor immune escape (Yang et al., 2021).

Although immunotherapies targeting T cells have shown promising clinical results, only a small percentage of patients have responded. The failure of monotherapy in many patients could be attributed to additional immune evasion mechanisms (Dempke et al., 2017). Notably, positive expression of PD-L1 in cervical and ectopic squamous cell carcinomas is often accompanied by the expression of TIM-3 and Gal-9, indicating the presence of checkpoint-based immune evasion mechanisms (Curley et al., 2020). Within the TIME, Gal-9 is found in nearly all immune cells and exhibits membranous expression in invasive and intraepithelial tumors. In invasive carcinomas, there is no significant difference in the expression of membranous TIM-3 or Gal-9 between cervical and vulvar locations. Furthermore, all PD-L1-positive squamous cell carcinomas co-express TIM-3 and Gal-9, suggesting the potential of combination therapies involving immune checkpoint inhibitors for future cervical cancer treatment. Exploring the interrelationship between different immune checkpoints may provide valuable insight in the fight against immune escape.

The use of Gal-9 ligands, such as lactose, can help regulate the inhibitory receptors PD-1 and TIM-3. Undoubtedly, new therapies centered around anti-Gal-9 will soon be proposed (Bailly et al., 2021). As an endoplasmic reticulum membrane-binding protein, stimulator of interferon genes (STING)’s activation of oligomers triggers downstream events that promote immune response activation (Yu et al., 2022). Recent findings by Shuang Zheng et al. (Zheng et al., 2023) have revealed an important mechanism by which Gal-9 mediates tumor immune escape through the STING/IFN-β signaling pathway. This, along with the research by Yang et al. (2021), suggests a novel combination strategy involving anti-Gal-9, including cases resistant to PD-1/PD- L1 blockade. It is worth noting that the clinical emergence of combination therapy involving Gal-9 and PD-L1 has shown promising results. Studies have demonstrated that the combination therapy of Gal-9 and PD-L1 can enhance the effectiveness of anti-PD-L1 antibody therapy in patients with pancreatic ductal adenocarcinoma (Li et al., 2023). In this context, lactose or its derivatives, as well as functionally equivalent products, may also hold potential utility. Not coincidentally, a recent study found that Gal-9 upregulation by anthracyclines is a novel mechanism mediating tumor immune escape, and Sun X et al. identified the combination of adriamycin and anti-Gal-9 therapies as a promising strategy for cancer treatment (Sun et al., 2023). Additionally, Choukrani G et al. found that gal-9 could be of therapeutic interest for (azacitidine-resistant) AML (Choukrani et al., 2023). The combination of Gal-9 with azacitidine induced more cell death compared to either azacitidine or Gal-9 alone.

## 6 Discussion

Gals have received increasing attention in the last decade due to their crucial role in tumorigenesis and cancer progression. This group of proteins exhibits diverse functionalities both inside and outside cells, leading to their varying impact on cancer development (Li et al., 2021). Among the Gals family, Gal-9 has been the focus of numerous studies, particularly regarding its role as a ligand for TIM-3 in various diseases.

It is worth mentioning that the literature we included in the previous section involved a Meta-analysis study of 4,166 HCC patients (An et al., 2021) and a multivariate analysis of 2,233 patients with multiple cancers (Irie et al., 2005; Yamauchi et al., 2006; Melief et al., 2017; Curley et al., 2020; Yuan et al., 2020), which clearly demonstrated the evidence for Gal-9 as an independent influencing factor in pan-cancer from an evidence-based medical perspective. Based on these evidences, we examined the relationship between Gal-9 and clinically common malignant hematologic and solid tumors. We explored the potential of Gal-9 as a predictive biomarker for the response to immune checkpoint blockade therapy, which holds promise for further clinical applications.

In fact, our study found that Gal-9 expression can be detected in different tumor tissues and cells, and most of the studies confirmed that there is a correlation between the variability of Gal-9 expression in tumors and the degree of malignancy of tumors. During the benign-to-malignant transition, a trend toward Gal-9 downregulation was demonstrated at both the RNA and protein levels. Studies have shown that detection of Gal-9 expression is superior to detection of lymph node invasion in diagnosing the presence of metastasis in breast cancer tissue specimens. In addition, patients with high Gal-9 expression had significantly higher survival than those with low Gal-9 expression, and similar results were seen in studies of melanoma, cervical squamous cell carcinoma, and hepatocellular carcinoma. These preliminary findings confirm that Gal-9 can play a role in tumor therapy and warrants further study in other cancers. For the study of the relationship between Gal-9 and tumors, in addition to exogenous interference with Gal-9 expression, Gal-9 protein itself can also be applied to target specific receptors or modulate tumor factor interactions, which should also take into account the different biological roles played by different Gal-9 isoforms.

An increasing number of studies are shedding light on the effects of Gal-9 on the immune components of TIME, and these studies aim to unravel the mechanisms by which Gal-9 coordinates the immune status of the body. Although it is difficult to establish a systematic framework of Gal-9’s impact on the tumor immune microenvironment as of the current relevant studies, these results provide ideas for future translation of Gal-9 into clinical research to some extent.

On the other hand, we highlight the potential of Gal-9-based combination immunotherapy in the clinic. In addition, Immune escape is an important hallmark of many tumors. We believe that Gal-9’s ability to counteract tumor cell evasion from immune surveillance may be influenced by tumor heterogeneity and its varying benefits for immunotherapy. Interestingly, studies on “unlimited proliferation” and “apoptosis”, also tumor hallmarks, have found that these two “hallmarks” are concentrated in gastrointestinal tumors (Tang et al., 2022), including colorectal cancers (Sasidharan Nair et al., 2018; Blair et al., 2021), EAC (Akashi et al., 2017), ESCC (Chiyo et al., 2019), gallbladder (Tadokoro et al., 2016), and bile duct (Kobayashi et al., 2015).

As previously mentioned, although the “Partnership” between Gal-9 and TIM-3 is being explored in cancer research, with studies on Gal-9 in malignant hematological diseases and solid tumors primarily focus on the Gal-9/TIM-3 pathway. Further investigations are needed to fully understand the versatility of Gal-9 itself in cancer. Gal-1, -3, -8, and -9, along with their respective sugar-carrying molecules, interact to inhibit antitumor responses and promote immunosuppression in specific cancer types. However, the antitumor effects of targeted Gal members do not solely rely on their downstream pathways. TIM-3, as a receptor for Gal-9, is widely expressed in immune cells, particularly T cells, while other immune checkpoints are also recognized by Gal-9. Therefore, exclusively focusing on the Gal-9/TIM-3 pathway in targeting studies may limit our understanding and overlook the independent properties of Gal-9 and TIM-3 themselves.

Considering the complex structures and functions of Gal-9 and TIM-3, it is possible that Gal-9 could serve as an effective independent therapeutic target in malignant hematological diseases and malignancies in the future. While the tumor microenvironment can be likened to a composite “soil,” different immune cells possess their own distinct characteristics. In this regard, we aim to establish a Gal-9-associated complex immune microenvironmental crosstalk network in the future by investigating the interactions of Gal-9 with a variety of immune cells.

Gal-9 has aroused great interest since its discovery. Early in the study, its characteristic function was that it could induce eosinophil aggregation and activation, and as the study continued, it also played a role in the regulation of cell differentiation, adhesion, apoptosis, inflammatory response, and anti-inhibition of rejection. Autoimmune diseases, inflammatory reactions, transplant rejection, and malignant blood diseases are all closely related to Gal-9. Even in recent studies, Gal-9 has been found to be involved in the antiviral response in diseases such as AIDS and COVID-19 (Iwasaki-Hozumi et al., 2023; Thorman et al., 2023). In recent years, more and more researchers have turned their attention to the multifaceted regulation of tumor cells by Gal-9: in various types of tumors, Gal-9 crosstalks with tumor cells by regulating apoptosis, metastasis, immune escape, and angiogenesis. Unfortunately, the regulation of tumorigenesis and development by Gal-9 is achieved through multiple mechanisms, and it is difficult to even summarize the commonality of the specific mechanisms of Gal-9 on tumors, except for the exploration of the Gal-9/TIM-3 pathway, and the application of Gal-9 to the clinic is yet to be further matured by systematic studies.

Given the intricate role of Gal-9 in different tumor types and within the tumor microenvironment, future research efforts should focus on exploring its upstream non-coding RNA and downstream target genes. Investigating these aspects will be crucial in understanding the molecular mechanisms associated with Gal-9 in tumor development. Additionally, bioengineering of recombinant Gal-9 has attracted interest in recent years, with a subset of reports suggesting that Gal-9 can interact homotypically and heterotypically with other members of the Gals in a CRD-dependent manner, and that such interactions can have enhanced affinity for the target. Furthermore, combining targeted Gal-9 therapy with immunotherapy or chemotherapy that involves checkpoint blockade may offer a potential treatment approach for tumors enriched with Gal-9. This synergistic approach has the potential to enhance treatment efficacy and improve outcomes for patients with Gal-9-associated tumors. In conclusion, the complex role that Gal-9 plays in tumors expands the knowledge of galactoglucan lectins, and although the role of this character is not clear from current studies, we cannot deny its potential in future tumor immunotherapy.

## Figures and Tables

**Table:** 

**Gal-9**	Galectin-9
**TIME**	tumor immune microenvironment
**PD-1**	programmed death-1
**PD-L1**	PD-Ligand-1
**Gals**	Galectins
**CRD**	carbohydrate recognition domain
**LAMP2**	lysosomal-associated membrane protein 2
**PDI**	protein disulfide bond isomerase
**LPS**	lipopolysaccharide
**Gal-1**	Galectin-1
**Gal-3**	Galectin-3
**Gal-8**	Galectin-8
**TAM**	tumor-associated macrophage
**APCs**	antigen-presenting cells
**Treg cells**	Regulatory T-cells
**N-CRD**	N-terminal Carbohydrate Recognition Domain
**C-CRD**	C-terminal CRD
**AML**	acute myeloid leukemia
**MDS**	Myelodysplastic syndromes
**TIM-3**	T cell immunoglobulin and mucin-domain containing-3
**VISTA**	V-domain Ig-containing suppressors of T cell activation
**NK cells**	natural killer cells
**TF**	treatment failure
**CR**	complete remission
**CLL**	chronic lymphocytic leukemia
**IFN-γ**	interferon-γ
**B-ALL**	B-cell acute lymphoblastic leukemia
**ALL**	acute lymphoblastic leukemia
**BLL**	B-lymphocytic leukemia
**CCK18**	cysteine-cleaved keratin 18
**OS**	overall survival
**DFS**	disease-free survival
**RFS**	recurrence-free survival
**TNBC**	triple-negative breast cancer
**TIL**	tumor-infiltrating lymphocyte
**SCLC**	small cell lung cancer
**NSCLC**	non-small cell lung cancer
**LCNEC**	lung large cell neuroendocrine carcinoma
**PSC**	pulmonary sarcomatoid carcinoma
**HCC**	hepatocellular carcinoma
**HBV**	chronic hepatitis B virus
**GC**	gastric cancer
**PPARγ**	peroxisome proliferator-activated receptor γ
**VEGFR-3**	vascular endothelial growth factor receptor 3
**IGF-1R**	insulin-like growth factor 1 receptor
**GB**	glioblastoma
**DCs**	dendritic cells
**EAC**	esophageal adenocarcinoma
**ESCC**	esophageal squamous cell carcinoma
**EGFR**	epidermal growth factor receptor
**IGF**	insulin-like growth factor
**FGFR3**	fibroblast growth factor receptor 3
**PCDC**	plasmacytoid dendritic cell-like macrophages
**MIBC**	muscle-invasive bladder cancer
**BMDM**	bone marrow-derived macrophages
**LPS**	lipopolysaccharide
**PTEN**	phosphatase and tensin homolog
**MCP-1**	monocyte chemotactic protein-1
**MDC**	macrophage-derived chemokines
**Th1 cells**	helper T cells 1
**Th2 cells**	helper T cells 2
**IgG1**	immunoglobulin G1
**STING**	stimulator of interferon genes

## References

[B1] Aanhane E. Schulkens I. A. Heusschen R. Castricum K. Leffler H. Griffioen A. W. (2018). Different angioregulatory activity of monovalent galectin-9 isoforms. Angiogenesis 21 (3), 545–555. 10.1007/s10456-018-9607-8 PubMed Abstract | 10.1007/s10456-018-9607-8 | Google Scholar 29500586

[B2] Abbas A. K. Murphy K. M. Sher A. (1996). Functional diversity of helper T lymphocytes. Nature 383 (6603), 787–793. 10.1038/383787a0 PubMed Abstract | 10.1038/383787a0 | Google Scholar 8893001

[B3] Akashi E. Fujihara S. Morishita A. Tadokoro T. Chiyo T. Fujikawa K. (2017). Effects of galectin-9 on apoptosis, cell cycle and autophagy in human esophageal adenocarcinoma cells. Oncol. Rep. 38 (1), 506–514. 10.3892/or.2017.5689 PubMed Abstract | 10.3892/or.2017.5689 | Google Scholar 28586026

[B4] An Y. Xu S. Liu Y. Xu X. Philips C. A. Chen J. (2021). Role of galectins in the liver diseases: a systematic review and meta-analysis. Front. Med. (Lausanne) 8, 744518. 10.3389/fmed.2021.744518 PubMed Abstract | 10.3389/fmed.2021.744518 | Google Scholar 34778306 PMC8578830

[B5] Asayama T. Tamura H. Ishibashi M. Kuribayashi-Hamada Y. Onodera-Kondo A. Okuyama N. (2017). Functional expression of Tim-3 on blasts and clinical impact of its ligand galectin-9 in myelodysplastic syndromes. Oncotarget 8 (51), 88904–88917. 10.18632/oncotarget.21492 PubMed Abstract | 10.18632/oncotarget.21492 | Google Scholar 29179486 PMC5687656

[B6] Ashraf G. M. Perveen A. Tabrez S. Jabir N. R. Damanhouri G. A. Zaidi S. K. (2015). Altered galectin glycosylation: potential factor for the diagnostics and therapeutics of various cardiovascular and neurological disorders. Adv. Exp. Med. Biol. 822, 67–84. 10.1007/978-3-319-08927-0_10 PubMed Abstract | 10.1007/978-3-319-08927-0_10 | Google Scholar 25416978

[B7] Ashraf G. M. Perveen A. Zaidi S. K. Ahmad A. Shakil S. Firoz C. K. (2017). Galectins-A potential target for cardiovascular therapy. Curr. Vasc. Pharmacol. 15 (4), 296–312. 10.2174/1570161115666170201113046 PubMed Abstract | 10.2174/1570161115666170201113046 | Google Scholar 28155611

[B8] Bacigalupo M. L. Manzi M. Rabinovich G. A. Troncoso M. F. (2013). Hierarchical and selective roles of galectins in hepatocarcinogenesis, liver fibrosis and inflammation of hepatocellular carcinoma. World J. Gastroenterol. 19 (47), 8831–8849. 10.3748/wjg.v19.i47.8831 PubMed Abstract | 10.3748/wjg.v19.i47.8831 | Google Scholar 24379606 PMC3870534

[B9] Bailly C. Thuru X. Quesnel B. (2021). Modulation of the gal-9/TIM-3 immune checkpoint with alpha-lactose. Does anomery of lactose matter? Cancers (Basel) 13 (24), 6365. 10.3390/cancers13246365 PubMed Abstract | 10.3390/cancers13246365 | Google Scholar 34944985 PMC8699133

[B10] Barondes S. H. Castronovo V. Cooper D. N. Cummings R. D. Drickamer K. Feizi T. (1994a). Galectins: a family of animal beta-galactoside-binding lectins. Cell 76 (4), 597–598. 10.1016/0092-8674(94)90498-7 PubMed Abstract | 10.1016/0092-8674(94)90498-7 | Google Scholar 8124704

[B11] Barondes S. H. Cooper D. N. Gitt M. A. Leffler H. (1994b). Galectins. Structure and function of a large family of animal lectins. J. Biol. Chem. 269 (33), 20807–20810. 10.1016/s0021-9258(17)31891-4 PubMed Abstract | 10.1016/s0021-9258(17)31891-4 | Google Scholar 8063692

[B12] Blair B. B. Funkhouser A. T. Goodwin J. L. Strigenz A. M. Chaballout B. H. Martin J. C. (2021). Increased circulating levels of galectin proteins in patients with breast, colon, and lung cancer. Cancers (Basel) 13 (19), 4819. 10.3390/cancers13194819 PubMed Abstract | 10.3390/cancers13194819 | Google Scholar 34638303 PMC8508020

[B13] Bray F. Ferlay J. Soerjomataram I. Siegel R. L. Torre L. A. Jemal A. (2018). Global cancer statistics 2018: GLOBOCAN estimates of incidence and mortality worldwide for 36 cancers in 185 countries. CA Cancer J. Clin. 68 (6), 394–424. 10.3322/caac.21492 PubMed Abstract | 10.3322/caac.21492 | Google Scholar 30207593

[B14] Cedeno-Laurent F. Dimitroff C. J. (2012). Galectins and their ligands: negative regulators of anti-tumor immunity. Glycoconj J. 29 (8-9), 619–625. 10.1007/s10719-012-9379-0 PubMed Abstract | 10.1007/s10719-012-9379-0 | Google Scholar 22544342 PMC3410977

[B15] Chabot S. Kashio Y. Seki M. Shirato Y. Nakamura K. Nishi N. (2002). Regulation of galectin-9 expression and release in Jurkat T cell line cells. Glycobiology 12 (2), 111–118. 10.1093/glycob/12.2.111 PubMed Abstract | 10.1093/glycob/12.2.111 | Google Scholar 11886844

[B16] Chaganty B. K. R. Qiu S. Gest A. Lu Y. Ivan C. Calin G. A. (2018). Trastuzumab upregulates PD-L1 as a potential mechanism of trastuzumab resistance through engagement of immune effector cells and stimulation of IFNγ secretion. Cancer Lett. 430, 47–56. 10.1016/j.canlet.2018.05.009 PubMed Abstract | 10.1016/j.canlet.2018.05.009 | Google Scholar 29746929 PMC6004098

[B17] Chaudhary S. Rawat S. Kulkarni A. Bilgrami A. L. Perveen A. Alghamdi B. S. (2022). Galectins-potential therapeutic targets for neurodegenerative disorders. Int. J. Mol. Sci. 23 (19), 11012. 10.3390/ijms231911012 PubMed Abstract | 10.3390/ijms231911012 | Google Scholar 36232314 PMC9569834

[B18] Che Y. Luo Z. Cao Y. Wang J. Xue Q. Sun N. (2022). Integrated pathological analysis to develop a Gal-9 based immune survival stratification to predict the outcome of lung large cell neuroendocrine carcinoma and its usefulness in immunotherapy. Int. J. Biol. Sci. 18 (15), 5913–5927. 10.7150/ijbs.76936 PubMed Abstract | 10.7150/ijbs.76936 | Google Scholar 36263183 PMC9576518

[B19] Chen P. Zhang L. Zhang W. Sun C. Wu C. He Y. (2020). Galectin-9-based immune risk score model helps to predict relapse in stage I-III small cell lung cancer. J. Immunother. Cancer 8 (2), e001391. 10.1136/jitc-2020-001391 PubMed Abstract | 10.1136/jitc-2020-001391 | Google Scholar 33082168 PMC7577067

[B20] Chen T. C. Chen C. H. Wang C. P. Lin P. H. Yang T. L. Lou P. J. (2017). The immunologic advantage of recurrent nasopharyngeal carcinoma from the viewpoint of Galectin-9/Tim-3-related changes in the tumour microenvironment. Sci. Rep. 7 (1), 10349. 10.1038/s41598-017-10386-y PubMed Abstract | 10.1038/s41598-017-10386-y | Google Scholar 28871094 PMC5583393

[B21] Chiyo T. Fujita K. Iwama H. Fujihara S. Tadokoro T. Ohura K. (2019). Galectin-9 induces mitochondria-mediated apoptosis of esophageal cancer *in vitro* and *in vivo* in a xenograft mouse model. Int. J. Mol. Sci. 20 (11), 2634. 10.3390/ijms20112634 PubMed Abstract | 10.3390/ijms20112634 | Google Scholar 31146370 PMC6600680

[B22] Cho S. J. Kook M. C. Lee J. H. Shin J. Y. Park J. Bae Y. K. (2015). Peroxisome proliferator-activated receptor gamma upregulates galectin-9 and predicts prognosis in intestinal-type gastric cancer. Int. J. Cancer 136 (4), 810–820. 10.1002/ijc.29056 PubMed Abstract | 10.1002/ijc.29056 | Google Scholar 24976296

[B23] Chou F. C. Chen H. Y. Kuo C. C. Sytwu H. K. (2018). Role of galectins in tumors and in clinical immunotherapy. Int. J. Mol. Sci. 19 (2), 430. 10.3390/ijms19020430 PubMed Abstract | 10.3390/ijms19020430 | Google Scholar 29389859 PMC5855652

[B24] Choukrani G. Visser N. Ustyanovska Avtenyuk N. Olthuis M. Marsman G. Ammatuna E. (2023). Galectin-9 has non-apoptotic cytotoxic activity toward acute myeloid leukemia independent of cytarabine resistance. Cell Death Discov. 9 (1), 228. 10.1038/s41420-023-01515-w PubMed Abstract | 10.1038/s41420-023-01515-w | Google Scholar 37407572 PMC10322858

[B25] Cristiani C. M. Turdo A. Ventura V. Apuzzo T. Capone M. Madonna G. (2019). Accumulation of circulating CCR7(+) natural killer cells marks melanoma evolution and reveals a CCL19-dependent metastatic pathway. Cancer Immunol. Res. 7 (5), 841–852. 10.1158/2326-6066.CIR-18-0651 PubMed Abstract | 10.1158/2326-6066.CIR-18-0651 | Google Scholar 30940644

[B26] Curley J. Conaway M. R. Chinn Z. Duska L. Stoler M. Mills A. M. (2020). Looking past PD-L1: expression of immune checkpoint TIM-3 and its ligand galectin-9 in cervical and vulvar squamous neoplasia. Mod. Pathol. 33 (6), 1182–1192. 10.1038/s41379-019-0433-3 PubMed Abstract | 10.1038/s41379-019-0433-3 | Google Scholar 32139873

[B27] Dama P. Tang M. Fulton N. Kline J. Liu H. (2019). Gal9/Tim-3 expression level is higher in AML patients who fail chemotherapy. J. Immunother. Cancer 7 (1), 175. 10.1186/s40425-019-0611-3 PubMed Abstract | 10.1186/s40425-019-0611-3 | Google Scholar 31291985 PMC6621946

[B28] D'Andrea A. Aste-Amezaga M. Valiante N. M. Ma X. Kubin M. Trinchieri G. (1993). Interleukin 10 (IL-10) inhibits human lymphocyte interferon gamma-production by suppressing natural killer cell stimulatory factor/IL-12 synthesis in accessory cells. J. Exp. Med. 178 (3), 1041–1048. 10.1084/jem.178.3.1041 PubMed Abstract | 10.1084/jem.178.3.1041 | Google Scholar 8102388 PMC2191152

[B29] Dempke W. C. M. Fenchel K. Uciechowski P. Dale S. P. (2017). Second- and third-generation drugs for immuno-oncology treatment-The more the better? Eur. J. Cancer 74, 55–72. 10.1016/j.ejca.2017.01.001 PubMed Abstract | 10.1016/j.ejca.2017.01.001 | Google Scholar 28335888

[B30] Elrod H. A. Sun S. Y. (2008). PPARgamma and apoptosis in cancer. PPAR Res. 2008, 704165. 10.1155/2008/704165 PubMed Abstract | 10.1155/2008/704165 | Google Scholar 18615184 PMC2442903

[B31] Enninga E. A. L. Chatzopoulos K. Butterfield J. T. Sutor S. L. Leontovich A. A. Nevala W. K. (2018). CD206-positive myeloid cells bind galectin-9 and promote a tumor-supportive microenvironment. J. Pathol. 245 (4), 468–477. 10.1002/path.5093 PubMed Abstract | 10.1002/path.5093 | Google Scholar 29732570 PMC6935353

[B32] Gajewski T. F. Schreiber H. Fu Y. X. (2013). Innate and adaptive immune cells in the tumor microenvironment. Nat. Immunol. 14 (10), 1014–1022. 10.1038/ni.2703 PubMed Abstract | 10.1038/ni.2703 | Google Scholar 24048123 PMC4118725

[B33] Gallazzi M. Ucciero M. A. M. Faraci D. G. Mahmoud A. M. Al Essa W. Gaidano G. (2022). New Frontiers in monoclonal antibodies for the targeted therapy of acute myeloid leukemia and myelodysplastic syndromes. Int. J. Mol. Sci. 23 (14), 7542. 10.3390/ijms23147542 PubMed Abstract | 10.3390/ijms23147542 | Google Scholar 35886899 PMC9320300

[B34] Giovannone N. Liang J. Antonopoulos A. Geddes Sweeney J. King S. L. Pochebit S. M. (2018). Galectin-9 suppresses B cell receptor signaling and is regulated by I-branching of N-glycans. Nat. Commun. 9 (1), 3287. 10.1038/s41467-018-05770-9 PubMed Abstract | 10.1038/s41467-018-05770-9 | Google Scholar 30120234 PMC6098069

[B35] Girnun G. D. Smith W. M. Drori S. Sarraf P. Mueller E. Eng C. (2002). APC-dependent suppression of colon carcinogenesis by PPARgamma. Proc. Natl. Acad. Sci. U. S. A. 99 (21), 13771–13776. 10.1073/pnas.162480299 PubMed Abstract | 10.1073/pnas.162480299 | Google Scholar 12370429 PMC129773

[B36] Gleason M. K. Lenvik T. R. McCullar V. Felices M. O'Brien M. S. Cooley S. A. (2012). Tim-3 is an inducible human natural killer cell receptor that enhances interferon gamma production in response to galectin-9. Blood 119 (13), 3064–3072. 10.1182/blood-2011-06-360321 PubMed Abstract | 10.1182/blood-2011-06-360321 | Google Scholar 22323453 PMC3321868

[B37] Goncalves Silva I. Yasinska I. M. Sakhnevych S. S. Fiedler W. Wellbrock J. Bardelli M. (2017). The tim-3-galectin-9 secretory pathway is involved in the immune escape of human acute myeloid leukemia cells. EBioMedicine 22, 44–57. 10.1016/j.ebiom.2017.07.018 PubMed Abstract | 10.1016/j.ebiom.2017.07.018 | Google Scholar 28750861 PMC5552242

[B38] Gu C. J. Wu H. Sheng C. y. Ni Q. c. (2013). Expression and prognostic value of galectin-9 in hepatocellular carcinoma patients. Zhonghua Yi Xue Za Zhi 93 (26), 2025–2028. 10.3760/cma.j.issn.0376-2491.2013.26.003 PubMed Abstract | 10.3760/cma.j.issn.0376-2491.2013.26.003 | Google Scholar 24169278

[B39] Guo H. Li B. Diao L. Wang H. Chen P. Jiang M. (2021). An immune-based risk-stratification system for predicting prognosis in pulmonary sarcomatoid carcinoma (PSC). Oncoimmunology 10 (1), 1947665. 10.1080/2162402X.2021.1947665 PubMed Abstract | 10.1080/2162402X.2021.1947665 | Google Scholar 34290908 PMC8279095

[B40] Hanahan D. Weinberg R. A. (2000). The hallmarks of cancer. Cell 100 (1), 57–70. 10.1016/s0092-8674(00)81683-9 PubMed Abstract | 10.1016/s0092-8674(00)81683-9 | Google Scholar 10647931

[B41] He Y. Jia K. Dziadziuszko R. Zhao S. Zhang X. Deng J. (2019). Galectin-9 in non-small cell lung cancer. Lung Cancer 136, 80–85. 10.1016/j.lungcan.2019.08.014 PubMed Abstract | 10.1016/j.lungcan.2019.08.014 | Google Scholar 31454748

[B42] Hirashima M. (2000). Ecalectin/galectin-9, a novel eosinophil chemoattractant: its function and production. Int. Arch. Allergy Immunol. 122 (Suppl. 1), 6–9. 10.1159/000053623 PubMed Abstract | 10.1159/000053623 | Google Scholar 10867499

[B43] Hirashima M. Kashio Y. Nishi N. Yamauchi A. Imaizumi T. a. Kageshita T. (2002). Galectin-9 in physiological and pathological conditions. Glycoconj J. 19 (7-9), 593–600. 10.1023/B:GLYC.0000014090.63206.2f PubMed Abstract | 10.1023/B:GLYC.0000014090.63206.2f | Google Scholar 14758084

[B44] Holderried T. A. W. de Vos L. Bawden E. G. Vogt T. J. Dietrich J. Zarbl R. (2019). Molecular and immune correlates of TIM-3 (HAVCR2) and galectin 9 (LGALS9) mRNA expression and DNA methylation in melanoma. Clin. Epigenetics 11 (1), 161. 10.1186/s13148-019-0752-8 PubMed Abstract | 10.1186/s13148-019-0752-8 | Google Scholar 31747929 PMC6868848

[B45] Irie A. Yamauchi A. Kontani K. Kihara M. Liu D. Shirato Y. (2005). Galectin-9 as a prognostic factor with antimetastatic potential in breast cancer. Clin. Cancer Res. 11 (8), 2962–2968. 10.1158/1078-0432.CCR-04-0861 PubMed Abstract | 10.1158/1078-0432.CCR-04-0861 | Google Scholar 15837748

[B46] Iwasaki-Hozumi H. Maeda Y. Niki T. Chagan-Yasutan H. Bai G. Matsuba T. (2023). Plasma N-cleaved galectin-9 is a surrogate marker for determining the severity of COVID-19 and monitoring the therapeutic effects of tocilizumab. Int. J. Mol. Sci. 24 (4), 3591. 10.3390/ijms24043591 PubMed Abstract | 10.3390/ijms24043591 | Google Scholar 36835000 PMC9964849

[B47] Jabbour E. Short N. J. Jain N. Haddad F. G. Welch M. A. Ravandi F. (2023). The evolution of acute lymphoblastic leukemia research and therapy at MD Anderson over four decades. J. Hematol. Oncol. 16 (1), 22. 10.1186/s13045-023-01409-5 PubMed Abstract | 10.1186/s13045-023-01409-5 | Google Scholar 36927623 PMC10018889

[B48] Jiao J. Jiao D. Yang F. Zhang J. Li Y. Han D. (2022). Galectin-9 expression predicts poor prognosis in hepatitis B virus-associated hepatocellular carcinoma. Aging (Albany NY) 14 (4), 1879–1890. 10.18632/aging.203909 PubMed Abstract | 10.18632/aging.203909 | Google Scholar 35202002 PMC8908941

[B49] Jin M. H. Nam A. R. Bang J. H. Oh K. S. Seo H. R. Kim J. M. (2021). WEE1 inhibition reverses trastuzumab resistance in HER2-positive cancers. Gastric Cancer 24 (5), 1003–1020. 10.1007/s10120-021-01176-7 PubMed Abstract | 10.1007/s10120-021-01176-7 | Google Scholar 33723720

[B50] Ju M. H. Byun K. D. Park E. H. Lee J. H. Han S. H. (2021). Association of galectin 9 expression with immune cell infiltration, programmed cell death ligand-1 expression, and patient's clinical outcome in triple-negative breast cancer. Biomedicines 9 (10), 1383. 10.3390/biomedicines9101383 PubMed Abstract | 10.3390/biomedicines9101383 | Google Scholar 34680500 PMC8533056

[B51] Kageshita T. Kashio Y. Yamauchi A. Seki M. Abedin M. J. Nishi N. (2002). Possible role of galectin-9 in cell aggregation and apoptosis of human melanoma cell lines and its clinical significance. Int. J. Cancer 99 (6), 809–816. 10.1002/ijc.10436 PubMed Abstract | 10.1002/ijc.10436 | Google Scholar 12115481

[B52] Kamradt T. Mitchison N. A. (2001). Tolerance and autoimmunity. N. Engl. J. Med. 344 (9), 655–664. 10.1056/NEJM200103013440907 PubMed Abstract | 10.1056/NEJM200103013440907 | Google Scholar 11228281

[B53] Kapetanakis N. I. Busson P. (2023). Galectins as pivotal components in oncogenesis and immune exclusion in human malignancies. Front. Immunol. 14, 1145268. 10.3389/fimmu.2023.1145268 PubMed Abstract | 10.3389/fimmu.2023.1145268 | Google Scholar 36817445 PMC9935586

[B54] Kashio Y. Nakamura K. Abedin M. J. Seki M. Nishi N. Yoshida N. (2003). Galectin-9 induces apoptosis through the calcium-calpain-caspase-1 pathway. J. Immunol. 170 (7), 3631–3636. 10.4049/jimmunol.170.7.3631 PubMed Abstract | 10.4049/jimmunol.170.7.3631 | Google Scholar 12646627

[B55] Kikushige Y. Miyamoto T. Yuda J. Jabbarzadeh-Tabrizi S. Shima T. Takayanagi S. i. (2015). A TIM-3/gal-9 autocrine stimulatory loop drives self-renewal of human myeloid leukemia stem cells and leukemic progression. Cell Stem Cell 17 (3), 341–352. 10.1016/j.stem.2015.07.011 PubMed Abstract | 10.1016/j.stem.2015.07.011 | Google Scholar 26279267

[B56] Knudsen A. M. Rudkjøbing S. J. Sørensen M. D. Dahlrot R. H. Kristensen B. W. (2021). Expression and prognostic value of the immune checkpoints galectin-9 and PD-L1 in glioblastomas. J. Neuropathol. Exp. Neurol. 80 (6), 541–551. 10.1093/jnen/nlab041 PubMed Abstract | 10.1093/jnen/nlab041 | Google Scholar 33990845

[B57] Kobayashi K. Morishita A. Iwama H. Fujita K. Okura R. Fujihara S. (2015). Galectin-9 suppresses cholangiocarcinoma cell proliferation by inducing apoptosis but not cell cycle arrest. Oncol. Rep. 34 (4), 1761–1770. 10.3892/or.2015.4197 PubMed Abstract | 10.3892/or.2015.4197 | Google Scholar 26260906

[B58] Kong F. Jin M. Cao D. Jia Z. Liu Y. Jiang J. (2020). Galectin-3 not Galectin-9 as a candidate prognosis marker for hepatocellular carcinoma. PeerJ 8, e9949. 10.7717/peerj.9949 PubMed Abstract | 10.7717/peerj.9949 | Google Scholar 32995093 PMC7501799

[B59] Lee M. Hamilton J. A. G. Talekar G. R. Ross A. J. Michael L. Rupji M. (2022). Obesity-induced galectin-9 is a therapeutic target in B-cell acute lymphoblastic leukemia. Nat. Commun. 13 (1), 1157. 10.1038/s41467-022-28839-y PubMed Abstract | 10.1038/s41467-022-28839-y | Google Scholar 35241678 PMC8894417

[B60] Li C. H. Hsu T. I. Chang Y. C. Chan M. H. Lu P. J. Hsiao M. (2021). Stationed or relocating: the seesawing EMT/MET determinants from embryonic development to cancer metastasis. Biomedicines 9 (9), 1265. 10.3390/biomedicines9091265 PubMed Abstract | 10.3390/biomedicines9091265 | Google Scholar 34572451 PMC8472300

[B61] Li E. Xu J. Chen Q. Zhang X. Xu X. Liang T. (2023). Galectin-9 and PD-L1 antibody blockade combination therapy inhibits tumour progression in pancreatic cancer. Immunotherapy 15 (3), 135–147. 10.2217/imt-2021-0075 PubMed Abstract | 10.2217/imt-2021-0075 | Google Scholar 36779368

[B62] Li H. Wu K. Tao K. Chen L. Zheng Q. Lu X. (2012). Tim-3/galectin-9 signaling pathway mediates T-cell dysfunction and predicts poor prognosis in patients with hepatitis B virus-associated hepatocellular carcinoma. Hepatology 56 (4), 1342–1351. 10.1002/hep.25777 PubMed Abstract | 10.1002/hep.25777 | Google Scholar 22505239

[B63] Liberal R. Grant C. R. Holder B. S. Ma Y. Mieli-Vergani G. Vergani D. (2012). The impaired immune regulation of autoimmune hepatitis is linked to a defective galectin-9/tim-3 pathway. Hepatology 56 (2), 677–686. 10.1002/hep.25682 PubMed Abstract | 10.1002/hep.25682 | Google Scholar 22371007

[B64] Liu C. Li Y. Xing X. Zhuang J. Wang J. Wang C. (2022a). Immunogenomic landscape analyses of immune molecule signature-based risk panel for patients with triple-negative breast cancer. Mol. Ther. Nucleic Acids 28, 670–684. 10.1016/j.omtn.2022.04.034 PubMed Abstract | 10.1016/j.omtn.2022.04.034 | Google Scholar 35614988 PMC9123090

[B65] Liu J. Yu Y. Liu C. Gao C. Zhuang J. Liu L. (2022c). Combinatorial regimens of chemotherapeutic agents: a new perspective on raising the heat of the tumor immune microenvironment. Front. Pharmacol. 13, 1035954. 10.3389/fphar.2022.1035954 PubMed Abstract | 10.3389/fphar.2022.1035954 | Google Scholar 36304169 PMC9593050

[B66] Liu K. Sun Q. Liu Q. Li H. Zhang W. Sun C. (2022b). Focus on immune checkpoint PD-1/PD-L1 pathway: new advances of polyphenol phytochemicals in tumor immunotherapy. Biomed. Pharmacother. 154, 113618. 10.1016/j.biopha.2022.113618 PubMed Abstract | 10.1016/j.biopha.2022.113618 | Google Scholar 36055113

[B67] Long B. Yu Z. Zhou H. Ma Z. Ren Y. Zhan H. (2018). Clinical characteristics and prognostic significance of galectins for patients with gastric cancer: a meta-analysis. Int. J. Surg. 56, 242–249. 10.1016/j.ijsu.2018.06.033 PubMed Abstract | 10.1016/j.ijsu.2018.06.033 | Google Scholar 29940258

[B68] Mani S. K. K. Andrisani O. (2018). Hepatitis B virus-associated hepatocellular carcinoma and hepatic cancer stem cells. Genes (Basel) 9 (3), 137. 10.3390/genes9030137 PubMed Abstract | 10.3390/genes9030137 | Google Scholar 29498629 PMC5867858

[B69] Marasco L. E. Kornblihtt A. R. (2023). The physiology of alternative splicing. Nat. Rev. Mol. Cell Biol. 24 (4), 242–254. 10.1038/s41580-022-00545-z PubMed Abstract | 10.1038/s41580-022-00545-z | Google Scholar 36229538

[B70] Meggyes M. Nagy D. U. Balassa T. Godony K. Peterfalvi A. Szereday L. (2021). Influence of galectin-9 treatment on the phenotype and function of NK-92MI cells in the presence of different serum supplements. Biomolecules 11 (8), 1066. 10.3390/biom11081066 PubMed Abstract | 10.3390/biom11081066 | Google Scholar 34439744 PMC8391477

[B71] Melief S. M. Visconti V. V. Visser M. van Diepen M. Kapiteijn E. H. W. van den Berg J. H. (2017). Long-term survival and clinical benefit from adoptive T-cell transfer in stage IV melanoma patients is determined by a four-parameter tumor immune signature. Cancer Immunol. Res. 5 (2), 170–179. 10.1158/2326-6066.CIR-16-0288 PubMed Abstract | 10.1158/2326-6066.CIR-16-0288 | Google Scholar 28073773

[B72] Morishita A. Nomura K. Tani J. Fujita K. Iwama H. Takuma K. (2021). Galectin-9 suppresses the tumor growth of colon cancer *in vitro* and *in vivo* . Oncol. Rep. 45 (6), 105. 10.3892/or.2021.8056 PubMed Abstract | 10.3892/or.2021.8056 | Google Scholar 33907832 PMC8072828

[B73] Mosmann T. R. Coffman R. L. (1989). TH1 and TH2 cells: different patterns of lymphokine secretion lead to different functional properties. Annu. Rev. Immunol. 7, 145–173. 10.1146/annurev.iy.07.040189.001045 PubMed Abstract | 10.1146/annurev.iy.07.040189.001045 | Google Scholar 2523712

[B74] Nagae M. Nishi N. Murata T. Usui T. Nakamura T. Wakatsuki S. (2006). Crystal structure of the galectin-9 N-terminal carbohydrate recognition domain from *Mus musculus* reveals the basic mechanism of carbohydrate recognition. J. Biol. Chem. 281 (47), 35884–35893. 10.1074/jbc.M606648200 PubMed Abstract | 10.1074/jbc.M606648200 | Google Scholar 16990264

[B75] Nagae M. Nishi N. Nakamura-Tsuruta S. Hirabayashi J. Wakatsuki S. Kato R. (2008). Structural analysis of the human galectin-9 N-terminal carbohydrate recognition domain reveals unexpected properties that differ from the mouse orthologue. J. Mol. Biol. 375 (1), 119–135. 10.1016/j.jmb.2007.09.060 PubMed Abstract | 10.1016/j.jmb.2007.09.060 | Google Scholar 18005988

[B76] Nobumoto A. Nagahara K. Oomizu S. Katoh S. Nishi N. Takeshita K. (2008). Galectin-9 suppresses tumor metastasis by blocking adhesion to endothelium and extracellular matrices. Glycobiology 18 (9), 735–744. 10.1093/glycob/cwn062 PubMed Abstract | 10.1093/glycob/cwn062 | Google Scholar 18579572

[B77] Nobumoto A. Oomizu S. Arikawa T. Katoh S. Nagahara K. Miyake M. (2009). Galectin-9 expands unique macrophages exhibiting plasmacytoid dendritic cell-like phenotypes that activate NK cells in tumor-bearing mice. Clin. Immunol. 130 (3), 322–330. 10.1016/j.clim.2008.09.014 PubMed Abstract | 10.1016/j.clim.2008.09.014 | Google Scholar 18974023

[B78] Okwor C. I. A. Oh J. S. Crawley A. M. Cooper C. L. Lee S. H. (2020). Expression of inhibitory receptors on T and NK cells defines immunological phenotypes of HCV patients with advanced liver fibrosis. iScience 23 (9), 101513. 10.1016/j.isci.2020.101513 PubMed Abstract | 10.1016/j.isci.2020.101513 | Google Scholar 32920488 PMC7492990

[B79] Pally D. Banerjee M. Hussain S. Kumar R. V. Petersson A. Rosendal E. (2022). Galectin-9 signaling drives breast cancer invasion through extracellular matrix. ACS Chem. Biol. 17 (6), 1376–1386. 10.1021/acschembio.1c00902 PubMed Abstract | 10.1021/acschembio.1c00902 | Google Scholar 35605245

[B80] Pang N. Alimu X. Chen R. Muhashi M. Ma J. Chen G. (2021). Activated Galectin-9/Tim3 promotes Treg and suppresses Th1 effector function in chronic lymphocytic leukemia. FASEB J. 35 (7), e21556. 10.1096/fj.202100013R PubMed Abstract | 10.1096/fj.202100013R | Google Scholar 34137463

[B81] Paul W. E. Seder R. A. (1994). Lymphocyte responses and cytokines. Cell 76 (2), 241–251. 10.1016/0092-8674(94)90332-8 PubMed Abstract | 10.1016/0092-8674(94)90332-8 | Google Scholar 7904900

[B82] Qi Y. Chang Y. Wang Z. Chen L. Kong Y. Zhang P. (2019). Tumor-associated macrophages expressing galectin-9 identify immunoevasive subtype muscle-invasive bladder cancer with poor prognosis but favorable adjuvant chemotherapeutic response. Cancer Immunol. Immunother. 68 (12), 2067–2080. 10.1007/s00262-019-02429-2 PubMed Abstract | 10.1007/s00262-019-02429-2 | Google Scholar 31720813 PMC11028176

[B83] Rahmati A. Bigam S. Elahi S. (2023). Galectin-9 promotes natural killer cells activity via interaction with CD44. Front. Immunol. 14, 1131379. 10.3389/fimmu.2023.1131379 PubMed Abstract | 10.3389/fimmu.2023.1131379 | Google Scholar 37006235 PMC10060867

[B84] Romagnani S. (1994). Lymphokine production by human T cells in disease states. Annu. Rev. Immunol. 12, 227–257. 10.1146/annurev.iy.12.040194.001303 PubMed Abstract | 10.1146/annurev.iy.12.040194.001303 | Google Scholar 8011282

[B85] Ruvolo P. P. (2019). Galectins as regulators of cell survival in the leukemia niche. Adv. Biol. Regul. 71, 41–54. 10.1016/j.jbior.2018.09.003 PubMed Abstract | 10.1016/j.jbior.2018.09.003 | Google Scholar 30245264

[B86] Sasidharan Nair V. Toor S. M. Taha R. Z. Shaath H. Elkord E. (2018). DNA methylation and repressive histones in the promoters of PD-1, CTLA-4, TIM-3, LAG-3, TIGIT, PD-L1, and galectin-9 genes in human colorectal cancer. Clin. Epigenetics 10 (1), 104. 10.1186/s13148-018-0539-3 PubMed Abstract | 10.1186/s13148-018-0539-3 | Google Scholar 30081950 PMC6080402

[B87] Sato M. Nishi N. Shoji H. Seki M. Hashidate T. Hirabayashi J. (2002). Functional analysis of the carbohydrate recognition domains and a linker peptide of galectin-9 as to eosinophil chemoattractant activity. Glycobiology 12 (3), 191–197. 10.1093/glycob/12.3.191 PubMed Abstract | 10.1093/glycob/12.3.191 | Google Scholar 11971863

[B88] Sher A. Coffman R. L. (1992). Regulation of immunity to parasites by T cells and T cell-derived cytokines. Annu. Rev. Immunol. 10, 385–409. 10.1146/annurev.iy.10.040192.002125 PubMed Abstract | 10.1146/annurev.iy.10.040192.002125 | Google Scholar 1590992

[B89] Street N. E. Mosmann T. R. (1991). Functional diversity of T lymphocytes due to secretion of different cytokine patterns. FASEB J. 5 (2), 171–177. 10.1096/fasebj.5.2.1825981 PubMed Abstract | 10.1096/fasebj.5.2.1825981 | Google Scholar 1825981

[B90] Su S. Zhao J. Xing Y. Zhang X. Liu J. Ouyang Q. (2018). Immune checkpoint inhibition overcomes ADCP-induced immunosuppression by macrophages. Cell 175 (2), 442–457. 10.1016/j.cell.2018.09.007 PubMed Abstract | 10.1016/j.cell.2018.09.007 | Google Scholar 30290143

[B91] Sun X. Wang W. J. Lang J. Yang R. Shen W. J. Sun L. (2023). Inhibition of Galectin-9 sensitizes tumors to anthracycline treatment via inducing antitumor immunity. Int. J. Biol. Sci. 19 (14), 4644–4656. 10.7150/ijbs.84108 PubMed Abstract | 10.7150/ijbs.84108 | Google Scholar 37781042 PMC10535704

[B92] Suszczyk D. Skiba W. Pawłowska A. Polak G. Tarkowski R. Wertel I. (2023). Expression of gal-9 on dendritic cells and soluble forms of TIM-3/gal-9 in patients suffering from endometriosis. Int. J. Mol. Sci. 24 (6), 5948. 10.3390/ijms24065948 PubMed Abstract | 10.3390/ijms24065948 | Google Scholar 36983021 PMC10056739

[B93] Tadokoro T. Morishita A. Fujihara S. Iwama H. Niki T. Fujita K. (2016). Galectin-9: an anticancer molecule for gallbladder carcinoma. Int. J. Oncol. 48 (3), 1165–1174. 10.3892/ijo.2016.3347 PubMed Abstract | 10.3892/ijo.2016.3347 | Google Scholar 26797414

[B94] Taghiloo S. Allahmoradi E. Ebadi R. Tehrani M. Hosseini-Khah Z. Janbabaei G. (2017). Upregulation of galectin-9 and PD-L1 immune checkpoints molecules in patients with chronic lymphocytic leukemia. Asian Pac J. Cancer Prev. 18 (8), 2269–2274. 10.22034/APJCP.2017.18.8.2269 PubMed Abstract | 10.22034/APJCP.2017.18.8.2269 | Google Scholar 28843266 PMC5697491

[B95] Takano J. Morishita A. Fujihara S. Iwama H. Kokado F. Fujikawa K. (2016). Galectin-9 suppresses the proliferation of gastric cancer cells *in vitro* . Oncol. Rep. 35 (2), 851–860. 10.3892/or.2015.4452 PubMed Abstract | 10.3892/or.2015.4452 | Google Scholar 26717877

[B96] Tang X. Y. Luo Z. L. Xiong Y. L. Yang J. Shi A. P. Zheng K. F. (2022). The proliferative role of immune checkpoints in tumors: double regulation. Cancers (Basel) 14 (21), 5374. 10.3390/cancers14215374 PubMed Abstract | 10.3390/cancers14215374 | Google Scholar 36358792 PMC9657406

[B97] Thorman J. Björkman P. Sasinovich S. Tesfaye F. Mulleta D. Medstrand P. (2023). Performance of galectin-9 for identification of HIV viremia in adults receiving antiretroviral therapy in a resource-limited setting. J. Acquir Immune Defic. Syndr. 93 (3), 244–250. 10.1097/QAI.0000000000003196 PubMed Abstract | 10.1097/QAI.0000000000003196 | Google Scholar 36961948

[B98] Tontonoz P. Spiegelman B. M. (2008). Fat and beyond: the diverse biology of PPARgamma. Annu. Rev. Biochem. 77, 289–312. 10.1146/annurev.biochem.77.061307.091829 PubMed Abstract | 10.1146/annurev.biochem.77.061307.091829 | Google Scholar 18518822

[B99] Wada J. Kanwar Y. S. (1997). Identification and characterization of galectin-9, a novel beta-galactoside-binding mammalian lectin. J. Biol. Chem. 272 (9), 6078–6086. 10.1074/jbc.272.9.6078 PubMed Abstract | 10.1074/jbc.272.9.6078 | Google Scholar 9038233

[B100] Wang M. Cai Y. Peng Y. Xu B. Hui W. Jiang Y. (2020b). Exosomal LGALS9 in the cerebrospinal fluid of glioblastoma patients suppressed dendritic cell antigen presentation and cytotoxic T-cell immunity. Cell Death Dis. 11 (10), 896. 10.1038/s41419-020-03042-3 PubMed Abstract | 10.1038/s41419-020-03042-3 | Google Scholar 33093453 PMC7582167

[B101] Wang R. Song S. Harada K. Ghazanfari Amlashi F. Badgwell B. Pizzi M. P. (2020a). Multiplex profiling of peritoneal metastases from gastric adenocarcinoma identified novel targets and molecular subtypes that predict treatment response. Gut 69 (1), 18–31. 10.1136/gutjnl-2018-318070 PubMed Abstract | 10.1136/gutjnl-2018-318070 | Google Scholar 31171626 PMC6943252

[B102] Wang Y. Zhao E. Zhang Z. Zhao G. Cao H. (2018). Association between Tim-3 and Gal-9 expression and gastric cancer prognosis. Oncol. Rep. 40 (4), 2115–2126. 10.3892/or.2018.6627 PubMed Abstract | 10.3892/or.2018.6627 | Google Scholar 30106451

[B103] Wiersma V. R. de Bruyn M. Helfrich W. Bremer E. (2013). Therapeutic potential of Galectin-9 in human disease. Med. Res. Rev. 33 (Suppl. 1), E102–E126. 10.1002/med.20249 PubMed Abstract | 10.1002/med.20249 | Google Scholar 21793015

[B104] Wiersma V. R. de Bruyn M. van Ginkel R. J. Sigar E. Hirashima M. Niki T. (2012). The glycan-binding protein galectin-9 has direct apoptotic activity toward melanoma cells. J. Invest. Dermatol 132 (9), 2302–2305. 10.1038/jid.2012.133 PubMed Abstract | 10.1038/jid.2012.133 | Google Scholar 22572821 PMC3422695

[B105] Wiersma V. R. de Bruyn M. Wei Y. van Ginkel R. J. Hirashima M. Niki T. (2015). The epithelial polarity regulator LGALS9/galectin-9 induces fatal frustrated autophagy in KRAS mutant colon carcinoma that depends on elevated basal autophagic flux. Autophagy 11 (8), 1373–1388. 10.1080/15548627.2015.1063767 PubMed Abstract | 10.1080/15548627.2015.1063767 | Google Scholar 26086204 PMC4590647

[B106] Yamauchi A. Kontani K. Kihara M. Nishi N. Yokomise H. Hirashima M. (2006). Galectin-9, a novel prognostic factor with antimetastatic potential in breast cancer. Breast J. 12 (5 Suppl. 2), S196–S200. 10.1111/j.1075-122X.2006.00334.x PubMed Abstract | 10.1111/j.1075-122X.2006.00334.x | Google Scholar 16959001

[B107] Yang J. Zhu L. Cai Y. Suo J. Jin J. (2014). Role of downregulation of galectin-9 in the tumorigenesis of gastric cancer. Int. J. Oncol. 45 (3), 1313–1320. 10.3892/ijo.2014.2494 PubMed Abstract | 10.3892/ijo.2014.2494 | Google Scholar 24919464

[B108] Yang R. Sun L. Li C. F. Wang Y. H. Yao J. Li H. (2021). Galectin-9 interacts with PD-1 and TIM-3 to regulate T cell death and is a target for cancer immunotherapy. Nat. Commun. 12 (1), 832. 10.1038/s41467-021-21099-2 PubMed Abstract | 10.1038/s41467-021-21099-2 | Google Scholar 33547304 PMC7864927

[B109] Yasinska I. M. Meyer N. H. Schlichtner S. Hussain R. Siligardi G. Casely-Hayford M. (2020). Ligand-receptor interactions of galectin-9 and VISTA suppress human T lymphocyte cytotoxic activity. Front. Immunol. 11, 580557. 10.3389/fimmu.2020.580557 PubMed Abstract | 10.3389/fimmu.2020.580557 | Google Scholar 33329552 PMC7715031

[B110] Yasinska I. M. Sakhnevych S. S. Pavlova L. Teo Hansen Selnø A. Teuscher Abeleira A. M. Benlaouer O. (2019). The tim-3-galectin-9 pathway and its regulatory mechanisms in human breast cancer. Front. Immunol. 10, 1594. 10.3389/fimmu.2019.01594 PubMed Abstract | 10.3389/fimmu.2019.01594 | Google Scholar 31354733 PMC6637653

[B111] Yoon H. K. Kim T. H. Park S. Jung H. Quan X. Park S. J. (2018). Effect of anthracycline and taxane on the expression of programmed cell death ligand-1 and galectin-9 in triple-negative breast cancer. Pathol. Res. Pract. 214 (10), 1626–1631. 10.1016/j.prp.2018.08.009 PubMed Abstract | 10.1016/j.prp.2018.08.009 | Google Scholar 30139555

[B112] Yoshikawa K. Ishida M. Yanai H. Tsuta K. Sekimoto M. Sugie T. (2022). Prognostic significance of the expression levels of T-cell immunoglobulin mucin-3 and its ligand galectin-9 for relapse-free survival in triple-negative breast cancer. Oncol. Lett. 23 (6), 197. 10.3892/ol.2022.13318 PubMed Abstract | 10.3892/ol.2022.13318 | Google Scholar 35572493 PMC9100485

[B113] Yu Y. Liu J. Liu C. Liu R. Liu L. Yu Z. (2022). Post-translational modifications of cGAS-STING: a critical switch for immune regulation. Cells 11 (19), 3043. 10.3390/cells11193043 PubMed Abstract | 10.3390/cells11193043 | Google Scholar 36231006 PMC9563579

[B114] Yuan F. Ming H. Wang Y. Yang Y. Yi L. Li T. (2020). Molecular and clinical characterization of Galectin-9 in glioma through 1,027 samples. J. Cell Physiol. 235 (5), 4326–4334. 10.1002/jcp.29309 PubMed Abstract | 10.1002/jcp.29309 | Google Scholar 31609000 PMC7028024

[B115] Zaheed M. Wilcken N. Willson M. L. O'Connell D. L. Goodwin A. (2019). Sequencing of anthracyclines and taxanes in neoadjuvant and adjuvant therapy for early breast cancer. Cochrane Database Syst. Rev. 2 (2), CD012873. 10.1002/14651858.CD012873.pub2 PubMed Abstract | 10.1002/14651858.CD012873.pub2 | Google Scholar 30776132 PMC6378927

[B116] Zhang Z. Y. Dong J. H. Chen Y. W. Wang X. Q. Li C. H. Wang J. (2012). Galectin-9 acts as a prognostic factor with antimetastatic potential in hepatocellular carcinoma. Asian Pac J. Cancer Prev. 13 (6), 2503–2509. 10.7314/apjcp.2012.13.6.2503 PubMed Abstract | 10.7314/apjcp.2012.13.6.2503 | Google Scholar 22938412

[B117] Zheng S. Song J. Linghu D. Yang R. Liu B. Xue Z. (2023). Galectin-9 blockade synergizes with ATM inhibition to induce potent anti-tumor immunity. Int. J. Biol. Sci. 19 (3), 981–993. 10.7150/ijbs.79852 PubMed Abstract | 10.7150/ijbs.79852 | Google Scholar 36778120 PMC9909994

[B118] Zheng Y. Feng W. Wang Y. J. Sun Y. Shi G. Yu Q. (2019). Galectins as potential emerging key targets in different types of leukemia. Eur. J. Pharmacol. 844, 73–78. 10.1016/j.ejphar.2018.11.019 PubMed Abstract | 10.1016/j.ejphar.2018.11.019 | Google Scholar 30452910

[B119] Zhou X. Sun L. Jing D. Xu G. Zhang J. Lin L. (2018). Galectin-9 expression predicts favorable clinical outcome in solid tumors: a systematic review and meta-analysis. Front. Physiol. 9, 452. 10.3389/fphys.2018.00452 PubMed Abstract | 10.3389/fphys.2018.00452 | Google Scholar 29765332 PMC5939667

[B120] Zhu C. Anderson A. C. Schubart A. Xiong H. Imitola J. Khoury S. J. (2005). The Tim-3 ligand galectin-9 negatively regulates T helper type 1 immunity. Nat. Immunol. 6 (12), 1245–1252. 10.1038/ni1271 PubMed Abstract | 10.1038/ni1271 | Google Scholar 16286920

